# Diversity and distribution of the lanthanome in aerobic methane-oxidising bacteria

**DOI:** 10.1186/s40793-025-00776-5

**Published:** 2025-09-29

**Authors:** Shamsudeen Umar Dandare, Alexander Allenby, Eleonora Silvano, Peter Nockemann, Yin Chen, Thomas J. Smith, Deepak Kumaresan

**Affiliations:** 1 School of Biological Sciences, Queen’s University Belfast, Belfast, BT9 5DL United Kingdom; 2 Faculty of Chemical and Life Sciences, Usmanu Danfodiyo University, Sokoto, Nigeria; 3 School of Life Sciences, University of Warwick, Coventry, CV4 7AL United Kingdom; 4 School of Biosciences, University of Birmingham, Birmingham, B15 2TT United Kingdom; 5 The QUILL Research Centre, School of Chemistry and Chemical Engineering, Queen’s University Belfast, Belfast, BT9 5AG United Kingdom; 6 Biomolecular Sciences Research Centre, Sheffield Hallam University, Sheffield, S1 1WB United Kingdom

**Keywords:** Lanthanides, Methanotrophs, Lanthanome, Methanol dehydrogenase, Horizontal gene transfer, Proteomics, Metatranscriptomics

## Abstract

**Background:**

Lanthanides (Ln) play important and often regulatory roles in the metabolism of methylotrophs, including methanotrophs, particularly through their involvement in methanol oxidation. However, the diversity, distribution, and ecological relevance of Ln-associated proteins (the lanthanome) in aerobic methane-oxidising bacteria (MOB) remain underexplored. This study investigates the lanthanome using genome, plasmid, and proteome data, alongside metatranscriptome data from methane-rich lake sediments.

**Results:**

We surveyed 179 genomes spanning Proteobacterial, Verrucomicrobial, and Actinobacterial MOBs to examine the distribution of Ln-dependent methanol dehydrogenases (MDHs) and Ln transport proteins. Distinct lineage-specific patterns were observed: XoxF5 was the most widespread MDH variant in Proteobacteria, while XoxF2 was restricted to Verrucomicrobia. Transporter systems also showed distinct patterns, with LanM restricted to Alphaproteobacteria, LanPepSY and LanA confined to Gammaproteobacteria, and LutH-like receptors broadly distributed across all lineages. Homologues of these genes were also detected on plasmids, indicating potential for horizontal gene transfer. In Lake Washington sediment metatranscriptomes, lanthanome transcripts were detected, with Proteobacteria as dominant contributors. Notably, a large fraction of *xoxF* transcripts were affiliated with non-MOB *Methylophilaceae*, consistent with known cooperative interactions with MOB. Using *Methylosinus trichosporium* OB3b as a model, we assessed methane oxidation and proteomic responses to soluble CeCl_3_ and a mixed-lanthanide ore. Lag phases were prolonged in the presence of lanthanides, particularly with ore, but methane oxidation rates converged across treatments after acclimation. Proteomic analysis revealed extensive condition-specific responses, with 724 proteins differentially expressed in Ore treatment compared to 60 under CeCl_3_. XoxF3 and XoxF5 were upregulated while MxaF and its accessory proteins were downregulated, consistent with the “lanthanide switch”. Notably, LanM was not expressed despite being encoded, whereas LutH-like receptor was downregulated under both treatments, likely reflecting regulatory control to prevent excess metal uptake. Additional upregulation of a TonB-dependent receptor and ABC transporter suggests a potential lanthanophore-mediated uptake strategy.

**Conclusion:**

This study highlights the diversity and ecological activity of Ln-binding and transport systems in MOBs, their plasmid localisation and potential mobility, and their distinct regulation under different Ln sources. The strong proteomic response to complex ore underscores the physiological flexibility of MOBs in coping with natural lanthanide forms. These findings provide a framework for ecological studies and candidate targets for biotechnological applications in methane bioconversion and sustainable lanthanide recovery from complex materials.

**Supplementary Information:**

The online version contains supplementary material available at 10.1186/s40793-025-00776-5.

## Introduction

The discovery in 2011 of bacteria capable of accumulating and utilising lanthanides (Ln) established the biological relevance of these rare earth elements (REE) in microbial metabolism. The first direct evidence of a biological role for Ln came from the identification of a lanthanoenzyme—an Ln-dependent methanol dehydrogenase (MDH) XoxF, found in several methylotrophs [1–3] and methanotrophs [4]. This enzyme catalyses the oxidation of methanol to formaldehyde and contains a pyrroloquinoline quinone (PQQ) redox cofactor that coordinates the Ln ion [5]. This discovery overturned the long-standing view that methanol oxidation was mediated exclusively by the calcium-dependent MDH MxaFI, a complex heterotetrameric enzyme, and introduced the simpler, homodimeric XoxF enzymes, which are now thought to be evolutionarily ancestral [6, 7]. While methylotrophs grow on reduced single-carbon compounds such as methanol, methanotrophs represent a specialised subset capable of oxidising methane. Aerobic methane-oxidising bacteria (MOB) initiate methane metabolism via methane monooxygenase (MMO), converting methane to methanol, which is then oxidised by MDH [5]. These organisms play a pivotal role in mitigating methane emissions, a potent greenhouse gas, and are gaining attention as microbial platforms in one-carbon-based biotechnologies [8, 9].

The presence of lanthanides modulates expression of XoxF via a regulatory mechanism known as the “lanthanide switch,” in which Ln availability represses *mxaFI* expression and induces *xoxF* transcription [10, 11]. XoxF proteins form multiple phylogenetic clades (e.g., XoxF1–5) that may differ in their biochemical properties, lanthanide preferences, and ecological roles. Despite this diversity, the biochemistry and physiological relevance of these clades remain underexplored, particularly in methanotrophs [6, 12, 13]. Moreover, while the role of lanthanides in XoxF function is well established, their involvement in other PQQ alcohol dehydrogenases has also been reported, including in methylotrophs [14, 15].

**Fig. 1:**
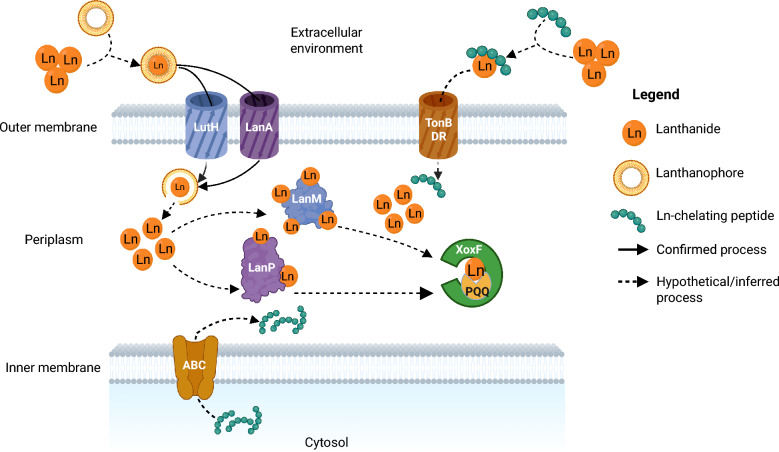
Conceptual model for lanthanide (Ln^3+^) uptake and intracellular trafficking in methanotrophic bacteria. Ln^3+^ ions or Ln–lanthanophore complexes are imported across the outer membrane via TonB-dependent receptors—LanA identified in Gammaproteobacteria, LutH-like in Alphaproteobacteria and a putative receptor (TonB-DR) identified in Methylosinus trichosporium OB3b. Certain strains may secrete Ln-chelating peptides via putative ABC transporters from the cytoplasm to the periplasm, and possibly to the extracellular environment through an unknown mechanism. The Ln-peptide complexes are then reclaimed through the putative TonB-DR. Together, the ABC transporter and TonB-DR function in a system that secretes Ln-chelating peptides and reclaims the resulting complexes, thereby trafficking extracellular Ln into the periplasm. In the periplasm, lanmodulin (LanM; primarily in Alphaproteobacteria) and LanPepSY (LanP; primarily in Gammaproteobacteria) bind and shuttle Ln^3+^ to target enzymes. The ions are ultimately incorporated into XoxF-type methanol dehydrogenases (MDHs) containing the pyrroloquinoline quinone (PQQ) cofactor. Solid arrows indicate experimentally supported processes, whereas dashed arrows represent hypothetical or inferred pathways, some of which have been confirmed in obligate methylotrophs. Blue-shaded proteins indicate those predominantly found in Alphaproteobacteria; purple-shaded proteins are mainly found in Gammaproteobacteria (Created in BioRender. Dandare, S. U. (2025) https://BioRender.com/v88jkpx)

The discovery and subsequent characterisation of lanthanoenzymes led to the identification of additional proteins involved in Ln sensing, acquisition, and transport, collectively referred to as the “lanthanome”. Cotruvo and colleagues identified lanmodulin (LanM), a highly selective Ln-binding protein in *Methylobacterium extorquens,* structurally similar to the calcium-binding protein calmodulin [16]. Two distinct TonB-dependent receptors—LanA and LutH—were later shown to be critical for the uptake of lanthanum and activation of the lanthanide switch in gammaproteobacterial [17] and alphaproteobacterial methanotrophs [18], respectively. The *lutH* gene is part of a recently characterised 10-gene *lut* cluster in *Methylorubrum extorquens* AM1 and the closely related phyllosphere bacterium PA1 [19, 20]. More recently, Hemmann and colleagues identified a 19 kDa periplasmic protein comprising two characteristic PepSY domains, named LanPepSY (LanP), in the obligate methylotroph *Methylobacillus flagellatus*, further expanding the known components of the lanthanome [21]. A schematic overview of the proposed lanthanide uptake and trafficking pathways in methanotrophic bacteria, highlighting key proteins, is presented in Fig. 1.

To date, most studies of the lanthanome have focused on methylotrophs, leaving the lanthanide-dependent physiology of methanotrophs underexplored. Given the ecological significance of aerobic MOB and their widespread distribution in terrestrial and aquatic environments, a broader understanding of lanthanide-associated traits in these organisms is needed. In particular, there is a need for comparative studies across the phylogenetic breadth of MOB, including type I (Gammaproteobacteria), type II (Alphaproteobacteria), and the more recently recognised Verrucomicrobia lineages. Linking this genomic diversity to ecological function and environmental adaptation will provide deeper insight into the roles that lanthanide-dependent systems play in methane cycling and microbial evolution.

In this study, we conducted a broad survey of aerobic MOB genomes to investigate the diversity and distribution of lanthanide-dependent enzymes and transporters. To assess the environmental relevance of these genes, we analysed metatranscriptomes from methane-rich lake sediments for their presence and expression in natural settings. Additionally, we screened all plasmids available in the Integrated Microbial Genomes (IMG) database to assess the potential for plasmid-associated horizontal gene transfer of lanthanide-associated traits. Finally, we performed growth assays and proteomic analyses to investigate how two differing lanthanide sources, pure cerium chloride (CeCl_3_) and a mixed lanthanide ore, affect methane oxidation and protein expression in the alphaproteobacterial model organism *Methylosinus trichosporium* OB3b.

## Methods

### MOB Genome selection, plasmid screening, and environmental metatranscriptome analysis

A total of 179 known aerobic MOB genomes were retrieved from the NCBI RefSeq database (accessed in October 2023) and concatenated to generate a custom MOB genome database. To construct this dataset, we performed a series of programmatic queries using NCBI’s Entrez system. First, microbial families known to include MOB were identified, and all descendant species and strains were retrieved from the NCBI Taxonomy database. These taxa were then used to query the NCBI Assembly database to collect all available genome assemblies.

We then filtered the results to retain only genomes accepted into RefSeq, excluding metagenome-assembled genomes (MAGs), low-quality, or anomalous assemblies. The resulting dataset was curated to exclude non-MOB organisms that are taxonomically assigned to MOB-containing families but lack evidence of methane oxidation, specifically the presence of key methanotrophy markers (*pmoA*, *mmoX*). This approach yielded a conservative but high-confidence dataset comprising exclusively verified isolate genomes of aerobic MOB.

The final custom database comprises 175 RefSeq-annotated genomes, three MAGs representing atmospheric methane oxidisers to ensure representation of this group, as no cultured isolates are available [22–24], and a recently characterised mycobacterial MOB that encodes the soluble methane monooxygenase (*mmoX*) [25]. All information regarding the custom database, including genome accession numbers, source environment, and genome completeness metrics (assessed using CheckM2 v1.1.0), is provided in Table S1.

A phylogenomic tree of MOB genomes was generated using the GToTree workflow (v1.7.00). The GToTree pipeline predicts genes using Prodigal (v2.6.3), searches for a panel of 74 bacterial single copy marker genes (SCGs) from genomes using HMMER3 (v3.3.2), aligns the retrieved genes with Muscle (v5.1.linux64), trims those alignments with trimAl (v1.4.rev15), concatenates the trimmed alignments and then performs phylogenetic reconstruction with FastTree (v2.1.11) [26–31].

To investigate the potential for plasmid-mediated dissemination of Ln-associated genes, we searched the IMG/PR database (https://img.jgi.doe.gov/cgi-bin/plasmid/main.cgi) [32] for plasmids. Specifically, we used BLASTP with reference sequences to retrieve *lanM*, *lanP*, and TonB-dependent receptors (*lanA* and *lutH-*like genes) from the plasmid database. This analysis was not restricted to methanotrophs, as our goal was to assess the broader taxonomic distribution of Ln-related transporters on plasmids and their potential role in horizontal gene transfer. To assess the expression of Ln-associated MOB-specific genes in the environment, we analysed the assembled metatranscriptomes of Lake Washington microbial communities (Study name: Freshwater Sediment Methanotrophic Microbial Communities from Lake Washington under Simulated Oxygen Tension) [33] available via the IMG portal. This dataset was selected because it provides high-resolution transcriptomic data from methane-enriched microcosms with a well-characterised community structure. Our objective was to perform a focused, proof-of-concept ecological assessment of Ln-associated gene expression in a relevant environmental context, rather than a broad global survey.

### Sequence similarity search, curation, and phylogenetic tree construction

In order to retrieve homologues for specific genes, we carried out searches (E-value cut off 1e-30) with specific hidden Markov model (HMM) profiles in the custom MOB genomes database using the HMMER tool (v3.4) [34]. HMM profiles from KOFAM [35] were used for MxaF and XoxF, and custom HMMs were developed for the remaining genes using RefSeq gene sequences. Homologues flagged by the HMM searches were retrieved from the MOB protein database by Seqkit (v2.9.0; [36]). The recovered sequences were then concatenated with curated reference sequences and were subsequently aligned using Clustal Omega (v1.2.4) with default parameters [37]. Conserved regions in the alignments were identified and trimmed using trimAl (v1.5. rev0) with the option –automated1 [27], and finally, a phylogenetic tree of the resulting aligned and trimmed sequences was constructed using FastTree (v2.1.11) with 1,000 replicates for bootstrap [28]. The phylogenetic trees were then used for manual curation of the retrieved homologues.

Homologues of specific Ln-dependent enzymes and transporters in plasmids and lake sediment metatranscriptomes databases were retrieved using the BLASTP (E-value cut off 1e-30; as described previously in [38]) within the IMG portal (Details on specific query sequences are included in Table S2). To remove any redundancy and for ease of analysis, candidate hits were clustered at 100% using CD-HIT [39]. Hits were curated for sequence length using CD-HIT, and sequences shorter than the specified length were discarded. Following clustering, downstream processing was carried out, as mentioned above, to build representative phylogenetic trees for each gene. Phylogenetic trees were visualised and annotated with metadata using the interactive tree of life (iTOL v5) tool [40].

For the genes with biochemically characterised motifs and domains, we analysed all the potential hits from the MOB genomes, plasmids, and metatranscriptomes to identify and *bona fide* sequences. Because each LanM protein contains four motifs corresponding to its EF-hand domains, we created a single regular expression (REGEX) pattern encompassing all four motifs. This pattern was then used to identify *bona fide* LanM sequences. A list of the motifs, REGEX patterns, and domains used in the search is provided in Table S3. The motif and domain analyses were done using Seqkit and the NCBI Conserved Domain Database (CDD), respectively.

### Growth experiments of ***Methylosinus trichosporium*** OB3b under CeCl_3_ and lanthanide ore treatments

*Methylosinus trichosporium* OB3b was initially cultured in Nitrate Mineral Salts (NMS) medium [41]. Cells were grown to the late exponential phase in NMS with 20% (v/v) methane (CH_4_), which was added to the headspace via injection through the septum. The cells were then harvested, washed twice, and transferred into dilute Nitrate Mineral Salts (DNMS) medium, prepared by tenfold dilution of NMS with ultrapure water, for growth experiments involving lanthanide treatments (Ce and ore). DNMS was specifically used in these experiments to minimise background metal concentrations. All media were prepared using ultrapure water (18 MΩ cm) obtained from an Elga Purelab Classic Life Science water purification system (Veolia Water Technologies, High Wycombe, UK). All experiments were conducted in 120 mL acid-washed glass serum vials, containing 20 mL of media, with a 100 mL headspace for the gases. Four different treatments were set up: (i) no added Ln, (ii) 25 μM CeCl_3_, (iii) 250 mg mixed Ln ore, and (iv) ore without cells (negative control). Each treatment was performed in three biological replicates and incubated at 30 °C with shaking (150 rpm) and with an initial headspace methane concentration of 20% (v/v). The concentration of CeCl_3_ was selected based on previous studies investigating the effect of lanthanides on methanotrophs [42–44]. The lanthanide ore used in this study is a pre-concentrate derived from a rare earth element (REE) deposit in Labrador/Newfoundland region of Canada. It is rich in lanthanide oxides, with cerium as the dominant component (Table S4), and contains traces of other elements such as aluminium (Al), niobium (Nb), and zirconium (Zr). While ICP and XRF analyses provided reliable quantification of the major REEs, trace elements (Al, Nb, and Zr) could not be accurately measured due to matrix complexity and detection limitations. We acknowledge that the REE concentrations in the Ore treatment are substantially higher than those typically encountered in natural environments where *M. trichosporium* OB3b may occur; however, this experimental setup was intended to probe the organism’s physiological response to a complex, less bioavailable Ln source and to explore its relevance to potential biotechnological applications such as biomining or biorecovery of Ln from geological materials. The optical density (OD_600_) of the cultures and CH_4_ concentration were measured daily to monitor growth and CH_4_ oxidation using a Clariostar microplate reader (BMG Labtech) and an Agilent 7890B system equipped with a flame ionisation detector, respectively. However, optical density measurements were attempted but could not be reliably obtained for the ore-treated cultures due to interference from suspended particles. For the gas chromatography, a FuSED-silica column (TG-5MS, 5% phenyl methylpolysiloxane) was used with helium (≥ 99.9% purity) as the carrier gas at a flow rate of 1.2 mL/min. Both the front inlet and detector were set at 300 °C, with an inlet pressure of 4.5 psi.

### Proteomics analysis

Cells were harvested at the end of the experiment through centrifugation, then resuspended in 1 mL of ddH_2_O and mixed with 200 μL 2X Laemmli buffer. Only three biological replicates were selected for each condition. Samples were boiled at 98 °C for 15 min, after which 30 μL was loaded onto a precast NuPAGE Bis–Tris gel (Invitrogen) and electrophoresed for 5 min at 200 V. The gel was stained with Coomassie Brilliant Blue and destained overnight in ddH_2_O. The stained region containing all proteins was excised, cut into small pieces, and subjected to in-gel tryptic digestion. Briefly, gel pieces were dehydrated in ethanol, reduced with 50 mM Tris-(2-carboxyethyl) phosphine hydrochloride, alkylated with 200 mM 2-chloroacetamide (CAA), and digested overnight at 37 °C using 2.5 ng/µL sequencing-grade trypsin (Roche).

Peptides were extracted using a formic acid-acetonitrile solution (5%:25%, v/v), dried in a speed vacuum concentrator, and resuspended in acetonitrile-trifluoroacetate (2%:0.1%, v/v) for the nanoLC-ESI–MS/MS run. Peptide separation was performed using an Ultimate 3000 RSLCnano (Dionex-LC Packings) equipped with two C18 columns: an Acclaim PepMap µ-precolumn cartridge (300 µm i.d. × 5 mm, 5 μm, 100 Å; Thermo Fisher Scientific) and a Bruker nanoElute Forty analytical column (75 µm × 40 cm, 1.9 µm). Mobile phase A consisted of 0.1% formic acid in water, and mobile phase B was 0.1% formic acid in acetonitrile. The gradient program was as follows: 4% to 25% B over 36 min, 25% to 35% B over 10 min, 35% to 90% B over 3 min, followed by a 10 min re-equilibration at 4% B. The flow rate was maintained at 350 nL min^−1^. The Ultimate 3000 RSLCnano was coupled online to a hybrid timsTOF Pro mass spectrometer (Bruker Daltonics, Germany) via a CaptiveSpray nano-electrospray ion source [45]. The instrument operated in Data-Dependent Parallel Accumulation-Serial Fragmentation (PASEF) mode. Peptides were separated by ion mobility according to their collisional cross sections and charge states. Acquisition settings were as follows: mass range 100–1700 m/z, ion mobility range 1/K_0_ (Start: 0.6 Vs/cm^2^; End: 1.6 Vs/cm^2^), ramp rate 9.42 Hz, and 100% duty cycle. Fragmentation spectra were acquired using PASEF, and peptide identification was performed by matching MS/MS spectra against the *M. trichosporium* OB3b protein database using the MaxQuant software package.

The label-free quantification (LFQ) was performed according to the framework described by Cox and Mann [46], with default parameters and the ‘match between runs' function enabled. Comparative proteomics analysis was performed using Perseus software (v1.6.5.0; MPI of Biochemistry). Statistical significance was assessed using a two-sample t-test, applying a false discovery rate (FDR) threshold of 0.01 and 0.05. While a log2 fold change of ± 2 is often used as a benchmark for biological relevance, in this study, all proteins that passed the FDR threshold were considered significantly differentially expressed, regardless of the magnitude of fold change. Only proteins present in all replicates of at least one condition were considered valid. The proteomics data have been deposited in the ProteomeXchange Consortium via the PRIDE [47] partner repository with the dataset identifier PXD063434.

The dataset was manually interrogated for changes in known lanthanide- and methanotrophy-related gene products. Functional assignment of gene products was performed using the KEGG Orthology (KO) database, based on the functional ortholog (K number) assignments of individual proteins in the IMG database.

## Results and discussion

**Fig. 2:**
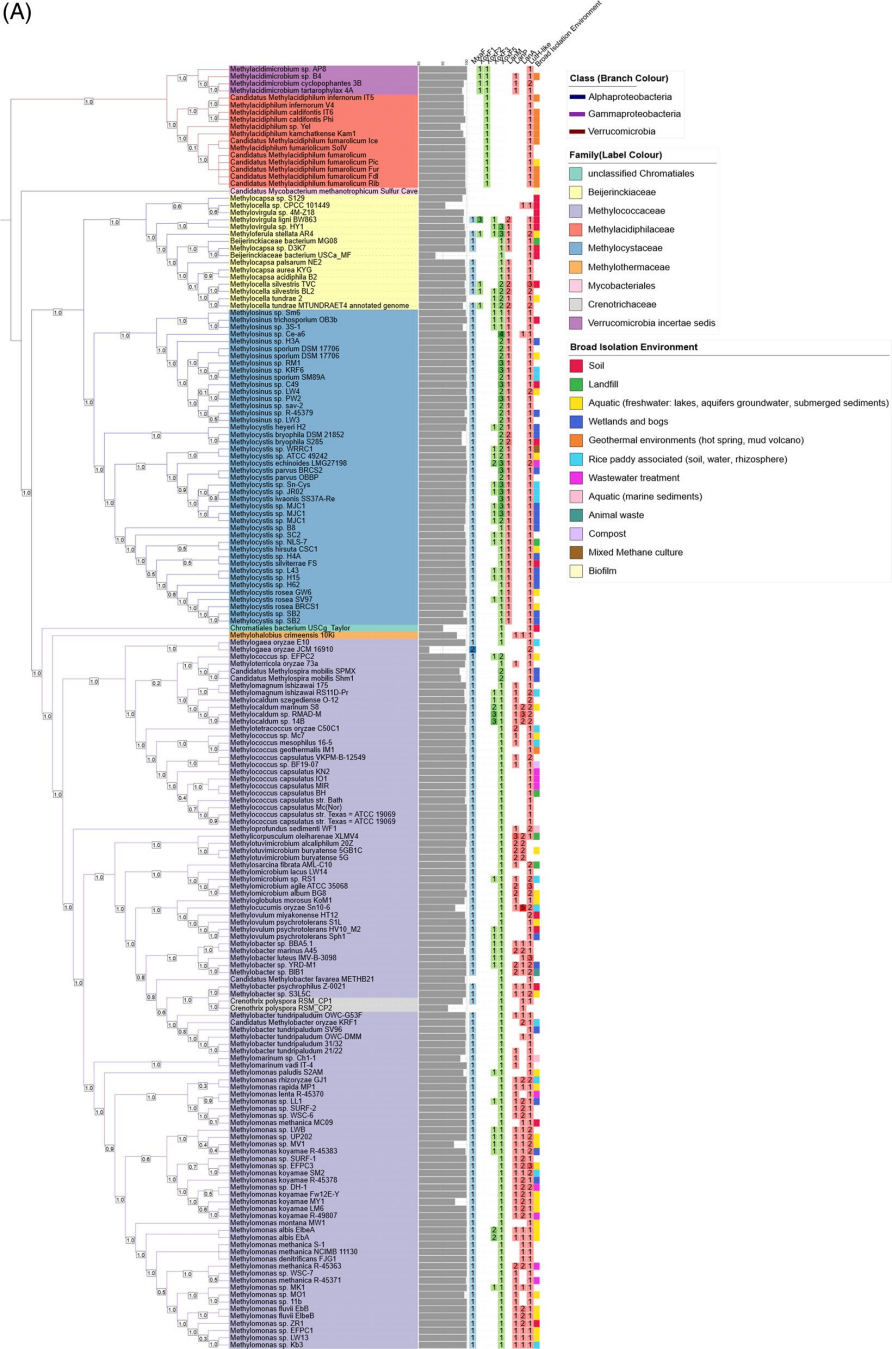

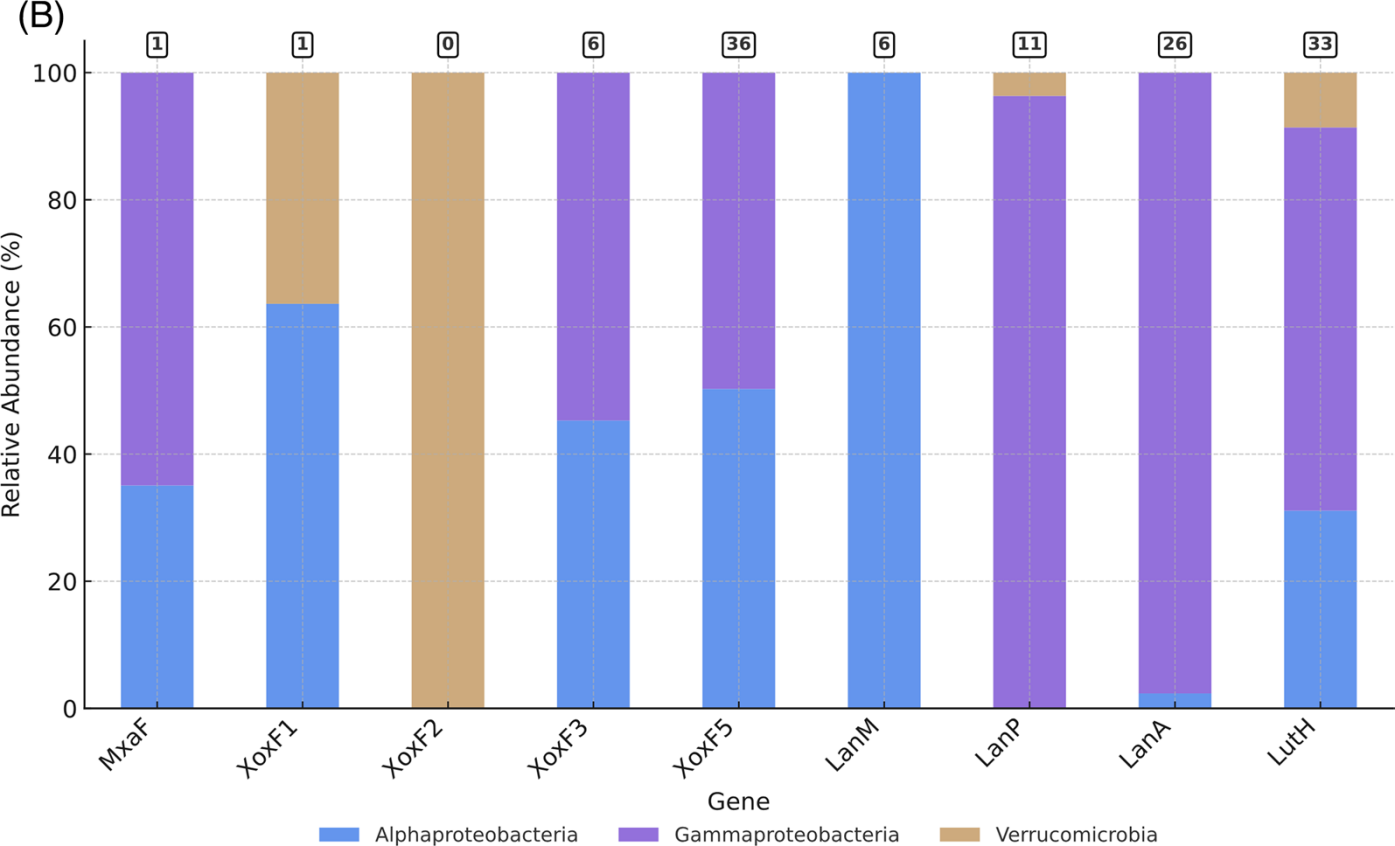
Phylogenomic distribution of methanol dehydrogenases and lanthanide-related genes in aerobic MOB. **A** Phylogenomic tree of 179 MOB genomes, constructed from single-copy marker genes and visualised using iTOL. Tree nodes are coloured by taxonomic class, and label colour ranges indicate bacterial families. The grey bar beside each genome represents genome completeness (%), while the colour strip shows the broad-scale environment of isolation. A heatmap shows the copy number of MxaF (blue), XoxF clades (green), and other lanthanome genes (red). The cells of the heatmap are shaded according to copy number, which is also displayed as text within each cell; cells with a copy number of zero were not rendered. Bootstrap support values (based on 1,000 replicates) are shown on nodes to one decimal place. **B** Stacked bar chart showing the distribution of each gene across major MOB taxonomic classes. Bars represent the percentage of genomes within each class containing the respective gene. The numbers in the squares on top of the bars show the number of genomes containing multiple copies of the gene

We performed a targeted survey of the “lanthanome” in aerobic MOBs using a custom database of 179 MOB genomes that included 101 Gammaproteobacteria, 60 Alphaproteobacteria, 17 Verrucomicrobia and 1 Actinomycete (Fig. 2a, Table S5). At the family level, the Methylococcaceae dominate with 97 representatives, while at the genus level, *Methylomonas* (39) show the highest representation, followed by *Methylocystis* (28). The key genes we surveyed encode the methanol dehydrogenases and known lanthanide transporter proteins (Lanmodulin, LanPepSY, LanA, and LutH), which together constitute the major known components of the lanthanome. We curated the hits to identify *bona fide* sequences that could be attributed to each functional class through phylogenetic trees, as well as domain and motif analyses.

### Distribution of methanol dehydrogenases in MOB genomes and sediment metatranscriptomes

Our genome-wide survey using key biomarker genes revealed the distribution pattern of variants of lanthanide-dependent (XoxF1, XoxF2, XoxF3, and XoxF5) and calcium-dependent methanol dehydrogenases (MxaF) (Table S5). A total of 175 out of the 179 MOB genomes in our database harboured at least one copy of the MDH, with several genomes possessing multiple copies of specific MDH variants, especially within the members of the *Proteobacteria* MOBs (Fig. 2). The four MOB genomes that do not have any canonical MDH include the recently characterised Actinobacterial MOB (*Candidatus M. methanotrophicum*): the only *Mycobacterium* shown to oxidise methane and 3 Alphaproteobacterial genomes (*Methylovirgula* sp. 4M-Z18, *Methylocapsa* sp. S129, and *Methylocella* sp. CPCC 101449) (Fig. 2a). All four genomes possess methane monooxygenase (MMO) genes (*pmoA* or *mmoX*), consistent with previous reports identifying them as methanotrophs. Although they lack canonical *mxaF* or *xoxF* genes, these organisms have been shown to use alternative methanol oxidation strategies. These include NAD-dependent MDH and other non-canonical oxidoreductases found in *Methylovirgula* and *Methylocapsa*, respectively [48]; or broad-substrate alcohol dehydrogenases in *Methylocella* [49] and *Candidatus M. methanotrophicum* [25], enabling them to oxidise methanol in the absence of classical MDH enzymes. The gammaproteobacterial *Methylogaea oryzae* JCM 16910 is the only organism in our database with two copies of *mxaF* but none of the *xoxF* variants.

A total of 154 *mxaF* and 288 *xoxF* hits, including multiple copies in single MOB genomes, were recovered, with the highest proportion of hits (71.9%) affiliated with *xoxF5* and the lowest (3.8%) affiliated with *xoxF1* (Fig. 2b). No *xoxF*4 variant was detected in MOB genomes, and it is well established that this clade occurs only in members within the family Methylophilaceae [12]. Most of the proteobacterial MOBs possess both *mxaF* and *xoxF5*, consistent with the findings of Huang and colleagues [50]. This further confirms the metabolic potential of methanotrophs to switch between using lanthanides or calcium, depending on the availability of either cofactor in their environment, commonly referred to as the “lanthanide switch”.

The *xoxF5* variant, the most prevalent and widely distributed variant among the *xoxF* genes in our survey, is harboured only by members of Proteobacterial MOBs (Fig. S1). Most of the Alphaproteobacterial MOB genomes (56 out of 60 genomes) harboured the *xoxF5* variant, with several genomes possessing more than one copy of the *xoxF5* variant, e.g. *Methylosinus* sp. Ce-a6 harbours four *xoxF5* variants, and 13 other MOB genomes, each possessing three copies of *xoxF5*. These 13 genomes belong to the genera *Methylocystis* (7), *Methylosinus* (4), *Methyloferulla* (1) and *Methylovirgula* (1). The *xoxF5* gene is widely distributed among methanotrophs and methylotrophs. Phylogenetic analysis revealed that these *xoxF5* sequences form several distinct clades, consistent with previous studies showing taxon-specific diversification within the *xoxF5* family reflecting functional and evolutionary divergence across taxa [12, 51]. In gammaproteobacterial MOBs, *xoxF5* was detected in all genomes except in *Methylogaea oryzae* JCM 16910. Only three gammaproteobacterial genomes (*Methylospira mobilis* Shm1, *Methylospira mobilis* SPMX and *Methylococcus* sp. EFPC2) harboured multiple copies of the *xoxF5* gene, with each genome possessing two copies only (Table S5). The multiple copies of *xoxF5* detected in the MOB genomes exhibit a patchy phylogenetic distribution pattern, with some copies clustering within the same clade and others grouping into distinct clades. This observed variation in the phylogenetic tree could provide insights into the potential evolutionary dynamics and divergent functional roles of the *xoxF5*. In genomes, such as *Methyloferula stellata* AR4 and *Methylovirgula* sp. HY1 that harbour three copies of *xoxF5,* all clustered within the same clade, suggesting recent duplication events and possible functional redundancy [52]. On the contrary, in other genomes, such as *Methylosinus* sp. Ce-a6 and *Methylosinus* sp. PW2, not all copies of *xoxF5* clustered together phylogenetically (Fig. S1), potentially suggesting more ancient duplication events and subsequent divergence. This divergence may reflect adaptation to varied ecological niches, cofactor preferences, or substrate specificities [6, 53].

The *xoxF*2 variant was only detected in verrucomicrobial MOBs (Fig. S2). In fact, all the Verrucomicrobiota harbour only XoxF2 as their MDH, except for four *Verrucomicrobiota incertae sedis* genomes (all belonging to the genus *Methylacidimicrobium*), which possess both XoxF1 and XoxF2 variants. This suggests functional specialisation of XoxF to these organisms, enabling them to metabolise methanol under distinct environmental conditions or with different cofactors.

XoxF1 was detected in five *Alphaproteobacteria* (three within the genus *Methylocella,* one *Methylovirgula* and one *Methylocystis*) isolated from acidic soils, and the only four *Verrucomicrobiota incertae sedis* genomes isolated from acidophilic/thermophilic environments [54]. The distribution of *xoxF1*, particularly in organisms present in acidic and high-temperature environments, suggests that the gene may confer ecological advantages to the organisms under such extreme conditions, playing a significant role in adaptation to pH and cofactor changes [4, 12]. Only the *Methylovirgula ligni* BW863 has multiple copies of *xoxF1*, possessing three copies, with two copies clustering together, while the other copy is grouped into a separate clade (Fig. S3).

Forty-five MOB genomes harboured the *xoxF3* variant that includes 23 Alphaproteobacteria and 22 Gammaproteobacteria MOB. Six genomes possessed multiple copies: one alphaproteobacterial MOB, *Methylocystis echinoides* LMG27198, and five gammaproteobacterial MOBs belonging to the genera *Methylocaldum* (3) and *Methylomonas* (2). Although the multiple copies in each genome cluster distinctly into separate clades, the genes from members of the same genus cluster together (Fig. S4). The lack of clustering of copies from the same organism suggests divergent evolution and possibly distinct functional roles of the different *xoxF3* copies within individual genomes. Moreover, the gene copies that cluster closely with those from other organisms of the same genus indicate that horizontal gene transfer (HGT) may have occurred. This is common in MOB as they often exchange genetic material to adapt to various environmental conditions [55].

**Fig. 3:**
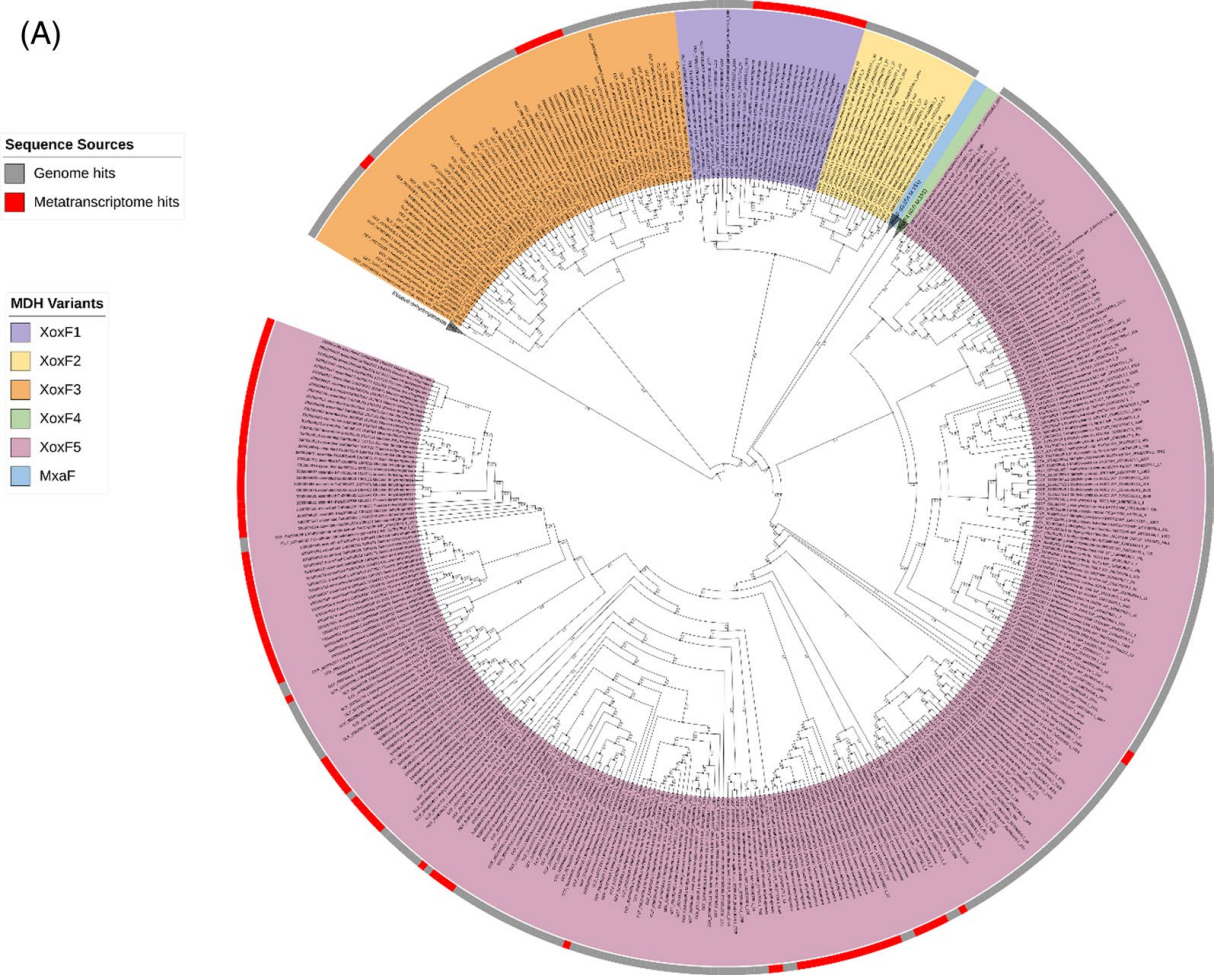

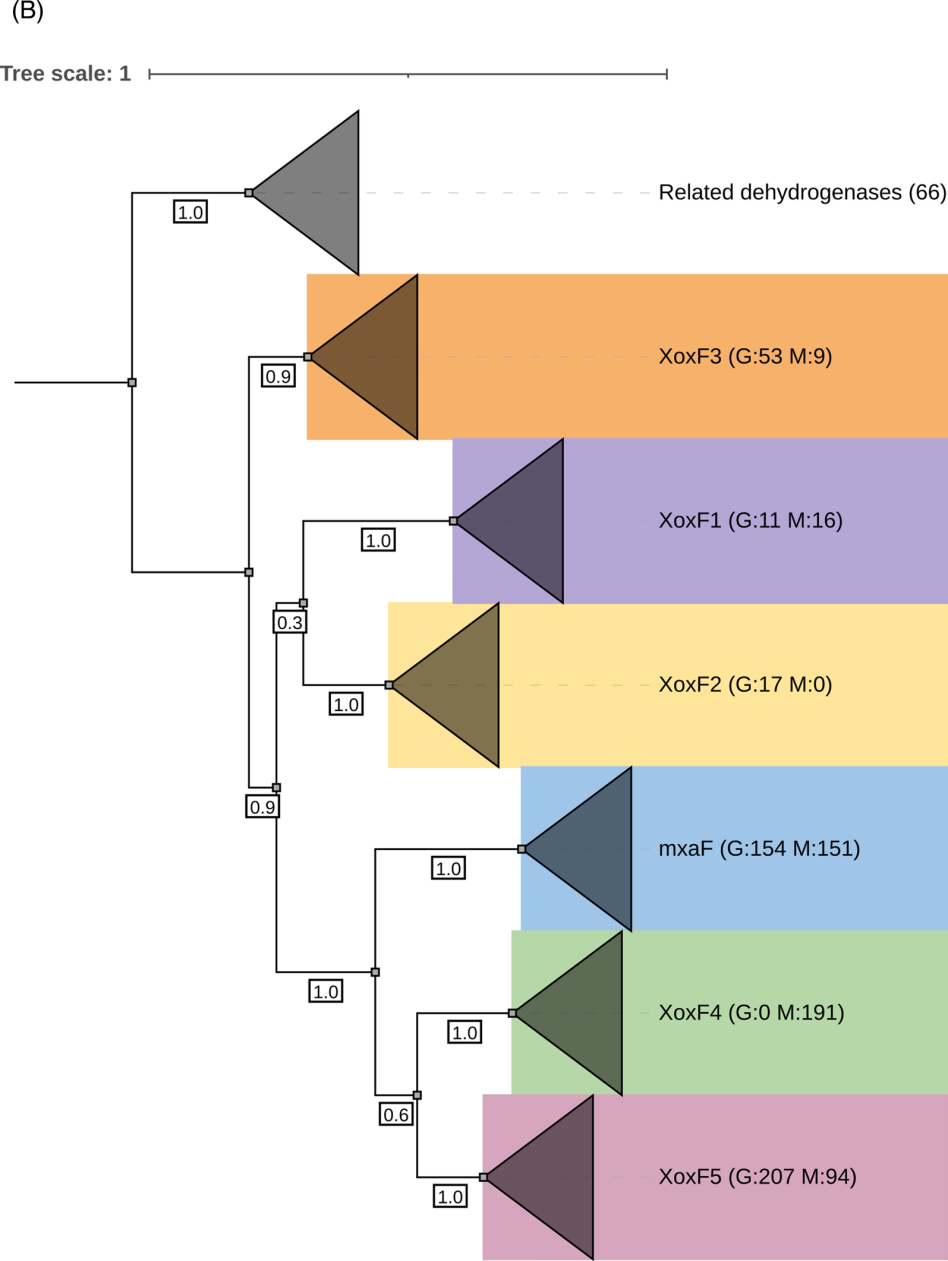
Distribution of MxaF and XoxF methanol dehydrogenases in MOB genomes and metatranscriptomes. **A** Circular phylogenetic tree showing XoxF sequences retrieved from 179 MOB genomes (grey colour strips) and from Lake Washington sediment metatranscriptomes (MTR, red colour strips). Sequences corresponding to MxaF, XoxF4, and related alcohol dehydrogenases were included in the analysis but are collapsed into single groups for clarity. **B** Rectangular phylogenetic tree with all clades collapsed. Numbers in parentheses indicate the number of sequences per clade. The letters **G** and **M** denote the number of genome and metatranscriptome hits, respectively. Coloured ranges indicate different XoxF variants. Bootstrap values (1,000 replicates) are shown on nodes to one decimal place. Trees were annotated using iTOL

To understand the distribution of *xoxF*s and *mxaF* in the natural environment, we analysed assembled metatranscriptomes from Lake Washington sediment microbial communities [33] for the presence of MDH transcripts. Although methane was supplied as the single carbon source, the resulting datasets include transcripts from both methanotrophs and methylotrophs that were transcriptionally active under the experimental conditions. A BLASTP search of the metatranscriptome returned over 6800 hits. However, most sequences were fragmented, so a length cutoff of ≥500 amino acids was applied to retain only Full-length or near Full-length MDH transcripts. A total of 535 transcripts were retained for the final phylogenetic analysis. All *xoxF* variants were detected in the metatranscriptomes except *xoxF2,* highlighting that MOB XoxF-MDHs are expressed and actively involved in the methane oxidation pathway, i.e. catalysing the oxidation of methanol to formaldehyde. However, more than 50% of the *xoxFs* identified in the metatranscriptomes were affiliated with the *xoxF4* clade, a group of MDH mainly found in non-MOBs (*Methylophilaceae*) (Fig. 3). This is unsurprising, giving that *Methylophilaceae* are ubiquitous in the environment, including in freshwater ecosystems [56]. Studies in Lake Washington have also provided evidence of a cooperative metabolic relationship between *Methylophilaceae* and MOB [57, 58]. The majority of *xoxF* metatranscripts are phylogenetically linked to MOB, particularly *Methylobacter*, a dominant MOB in freshwater environments. Notably, among the 94 *xoxF5* metatranscripts identified, only two (~2% of the *xoxF5* retained for analysis, both assigned to *Burkholderiales*) were not associated with MOB.

Although our primary plasmid analysis focused on Ln-associated transporters (LanM, LanP, LanA, and LutH), a preliminary BLASTP search of the IMG plasmid protein database revealed *xoxF*-like sequences on several plasmids from diverse taxa, including methylotrophs (data not shown). This suggests that the capacity for Ln-dependent methanol oxidation may, in some cases, be plasmid-encoded.

Overall, our genome and metatranscriptome survey highlights the diverse distribution patterns and evolutionary dynamics of methanol dehydrogenases in MOBs, with clear lineage-specific preferences for particular MDH variants. By classifying the source environments of the genomes using metadata from NCBI, we observed recurring associations between specific MDH types and broad habitat categories. For instance, *xoxF5* was prevalent among proteobacteria from aquatic and terrestrial environments, *xoxF2* was largely restricted to Verrucomicrobiota from geothermal habitats, and *xoxF1* appeared in some organisms from acidophilic or thermophilic environments. These associations suggest that different MDHs may be ecologically specialised, potentially reflecting functional adaptation to metal availability or environmental conditions.

### Distribution of lanthanide transporters in MOB genomes, plasmids, and metatranscriptomes

The lanthanide-binding and transport proteins also show interesting patterns. While the lanthanide-binding protein LanM is only present in *Alphaproteobacteria*, the TonB-dependent receptor LanA and the Ln-binding protein LanPepSY were primarily found in the gammaproteobacterial MOB genomes. The LutH-like protein (also a TonB-dependent receptor) is more widely distributed and abundant than any other lanthanome candidate investigated, occurring across all MOB phyla (Fig. 2b). The presence or absence of specific transporters may have important metabolic implications. Organisms encoding LanM, LanP or LanA may have more efficient or specific mechanisms for lanthanide acquisition, potentially enabling faster or more robust activation of lanthanide-dependent enzymes such as XoxFs [1, 4]. Conversely, the widespread distribution of the LutH-like receptor suggests it may represent a more generalist or basal lanthanide uptake route. These differences in transport machinery may influence how effectively organisms can scavenge scarce lanthanides from their environment, ultimately affecting their ability to thrive in metal-limited niches. Thus, the diversity of lanthanide transporters across MOBs may contribute to ecological fitness and niche differentiation in lanthanide-variable environments [4, 13].

LanA from *Methylotuvimicrobium buryatense* [17] and CQW49_RS02145, a protein recently found in *Methylosinus trichosporium* OB3b and homologous to the LutH of *Methylorubrum extorquens* AM1 [18], are the only characterised lanthanide transport proteins identified in MOBs to date.

**Fig. 4:**
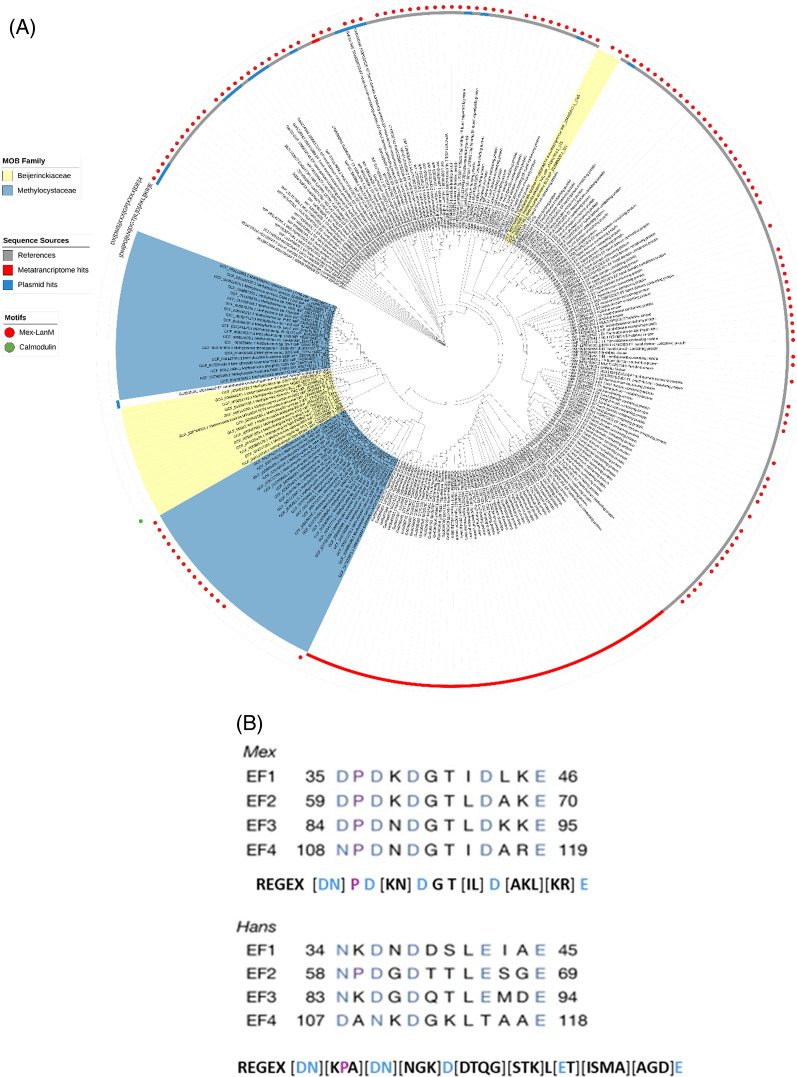
Distribution and diversity of Lanmodulin (LanM) in MOB genomes, plasmids, and metatranscriptomes. **A** Circular phylogenetic tree showing LanM sequences identified from MOB genomes (label coloured ranges), plasmids (blue strips), lake sediment metatranscriptomes (red strips), and reference sequences (grey strips). Red circles indicate bona fide LanM sequences matching the *Mex*-LanM motif, while the green circle represents a sequence with a calmodulin-like EF-hand motif. Bootstrap values (1,000 replicates) are shown on nodes to one decimal place. **B** The Mex- and Hans-LanM motifs from the biochemically characterised proteins [59]

Lanmodulin (LanM) was the first lanthanide-selective chelator to be discovered in *M. extorquens*, and it provided valuable insights into how Ln are selectively recognised and transported in methylotrophs [16]. Although it is similar to the calcium-binding protein calmodulin, as it possesses metal-binding EF hands, LanM was shown to uniquely respond to picomolar concentrations of all Ln^3+^ while responding to Ca^2+^ at millimolar concentrations–a remarkable 100-million-fold selectivity for Ln^3+^ over Ca^2+^ [16]. Our genome-wide survey detected the presence of LanM only in the MOB genomes belonging to the families Methylocystaceae and Beijerinkiaceae, which is consistent with the findings of Mattocks and colleagues [59]. We also identified 19 LanM sequences located on plasmids, including one from a methanotroph (*Methylocystis* sp. MGA3063736.1) and 18 from methylotrophs, comprising 15 from *Bradyrhizobium*, one from Mesorhizobium sp., and one each from *Methylobacterium nodulans* and *Methylorubrum aminovorans* (Fig. 4a). The presence of this gene in plasmids suggests its potential role in the survival and adaptation of these organisms to specific environments, as well as the potential for horizontal transfer of the gene to other bacteria. Further support for horizontal gene transfer (HGT) comes from our phylogenetic analysis of Lanmodulin (LanM) proteins, which revealed that three *Methylocella* strains (family Beijerinckiaceae, class Alphaproteobacteria) formed a well-supported clade with a homolog from *Derxia lacustris*, a member of the Betaproteobacteria (Fig. 4a). This unexpected grouping across distinct bacterial classes suggests phylogenetic incongruence, a hallmark of HGT. In addition, a BLAST search of the *Derxia* LanM sequence revealed moderate similarity (67% identity) to *Methylobacterium*, an alphaproteobacterium, further suggesting that homologous *lanM* sequences are shared across distant methylotrophic lineages. Together, the plasmid localisation and phylogenetic patterns support the hypothesis that LanM may be subject to horizontal gene transfer across taxonomically diverse methanotrophic and methylotrophic lineages.

Due to the close sequence relatedness of LanM and calmodulin, we performed a motif analysis to distinguish *bona fide* sequences from potential false positives. We used the identified motifs of the two biochemically characterised LanM from *M. extorquens* (*Mex*) and *Hansschlegelia quercus* (*Hans*) and created a single regular expression (REGEX) pattern (Fig. 4b) for identifying LanM sequences. These motifs are functionally significant, as they are associated with lanthanide binding and are essential for LanM activity [59, 60]. The motif analysis revealed that 17 MOB genome sequences (2 *Methylocella* and 15 *Methylocystis*) contained motifs matching *Mex-*LanM, supporting their identification as *bona fide* LanM and suggesting that LanM variants in MOBs are more similar to the *Mex*-type. At the same time, only one metatranscript was identified to encode a *bona fide* LanM (Fig. 4a), and no sequences matched the *Hans-*LanM. We also screened for the canonical bacterial EF-hand calmodulin motif (Table S3) to identify potential false positives; only one sequence from *Methylovirgula ligni* BW863 was detected (Fig. 4a). Sequences lacking known motifs may represent LanM or calmodulin variants with novel or divergent features not captured by the Mex-LanM or the calmodulin EF-hand motifs used in this analysis.

**Fig. 5:**
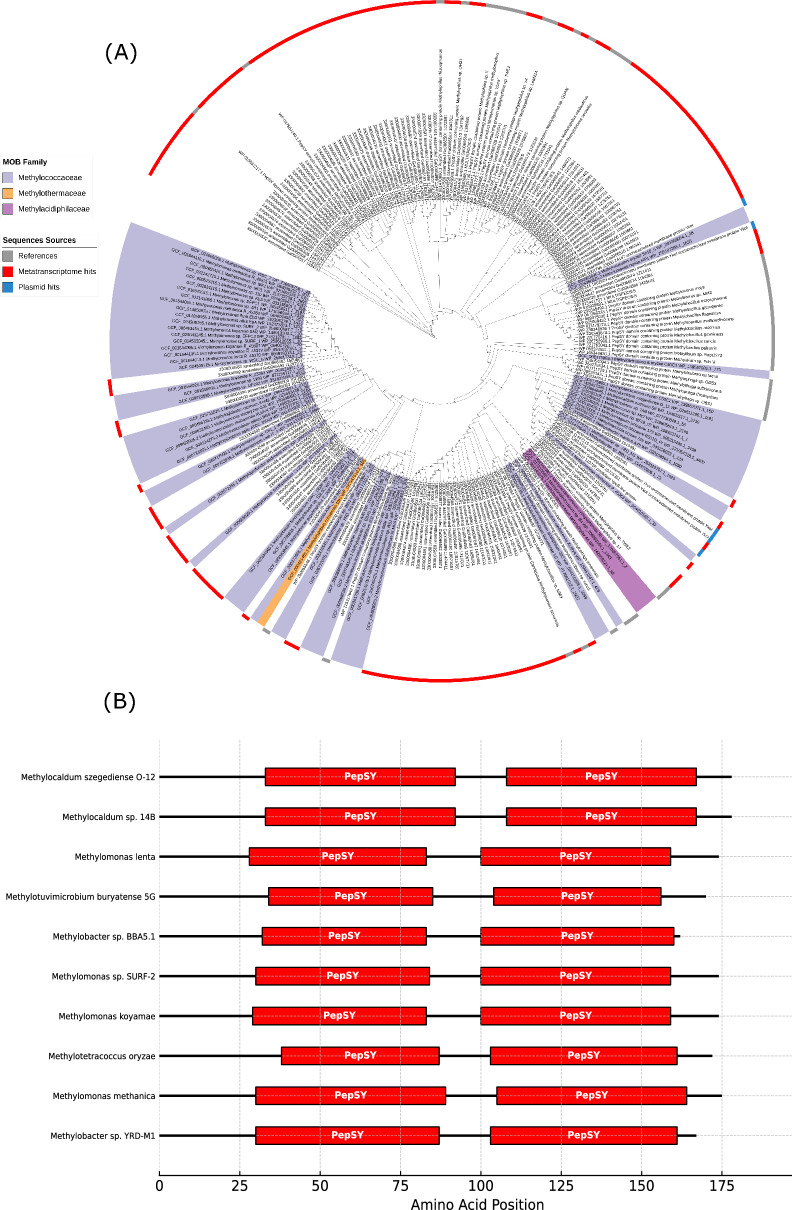
LanPepSY (LanP) in MOB genomes, plasmids and metatranscriptomes. **A** Circular phylogenetic tree of LanP sequences identified in MOB genomes (coloured ranges), plasmids (blue strips), lake sediment metatranscriptomes (red strips), and reference sequences (grey strips). The tree was annotated using iTOL. Coloured ranges indicate bacterial families. Bootstrap support values (based on 1000 replicates) are shown on nodes to one decimal place. **B** Representative MOB LanP sequences showing the two characteristic PepSY domains

LanPepSY (LanP), a lanthanide-binding protein, was recently discovered in the obligate methylotroph *Methylobacillus flagellatus*. Characterised by two PepSY domains, LanP is the first member of the PepSY family shown to bind lanthanides [21]. Our survey (Fig. 5a) revealed that LanP was predominantly detected within Methylococcales genomes belonging to the family *Methylococcaceae*, with a single hit identified in *Methylothermaceae* (*Methylohalobius crimeensis*). Additionally, five plasmid hits within the *Methylococcaceae* (*Methylomonas* sp., *Methylomicrobium* sp., *Methylobacter* sp., and *Methylomonas methanica*) were found to encode LanP. In Lake sediment metatranscriptomes, we recovered 406 sequences, confirming their active expression in this environment. After curation based on length cut-off and clustering to reduce redundancy, 132 sequences remained and were used for construction of the phylogenetic tree.

To verify the authenticity of the retrieved LanP sequences, we searched for the PepSY domain in all 249 curated sequences (from genomes, metatranscriptomes, and plasmids) using the Conserved Domain Database (CDD). All sequences contained the two characteristic PepSY domains, each approximately 60 amino acids in length, confirming their identity as *bona fide* LanP proteins (Fig. 5b). Although LanP appears taxonomically restricted to a few MOB families, the genomes encoding it were isolated from diverse environments including agricultural soils, aquatic sediment, and thermal habitats. Its expression in a single lake water metatranscriptome suggests that it is actively expressed in natural ecosystems under certain environmental conditions, supporting its potential ecological relevance.

**Fig. 6:**
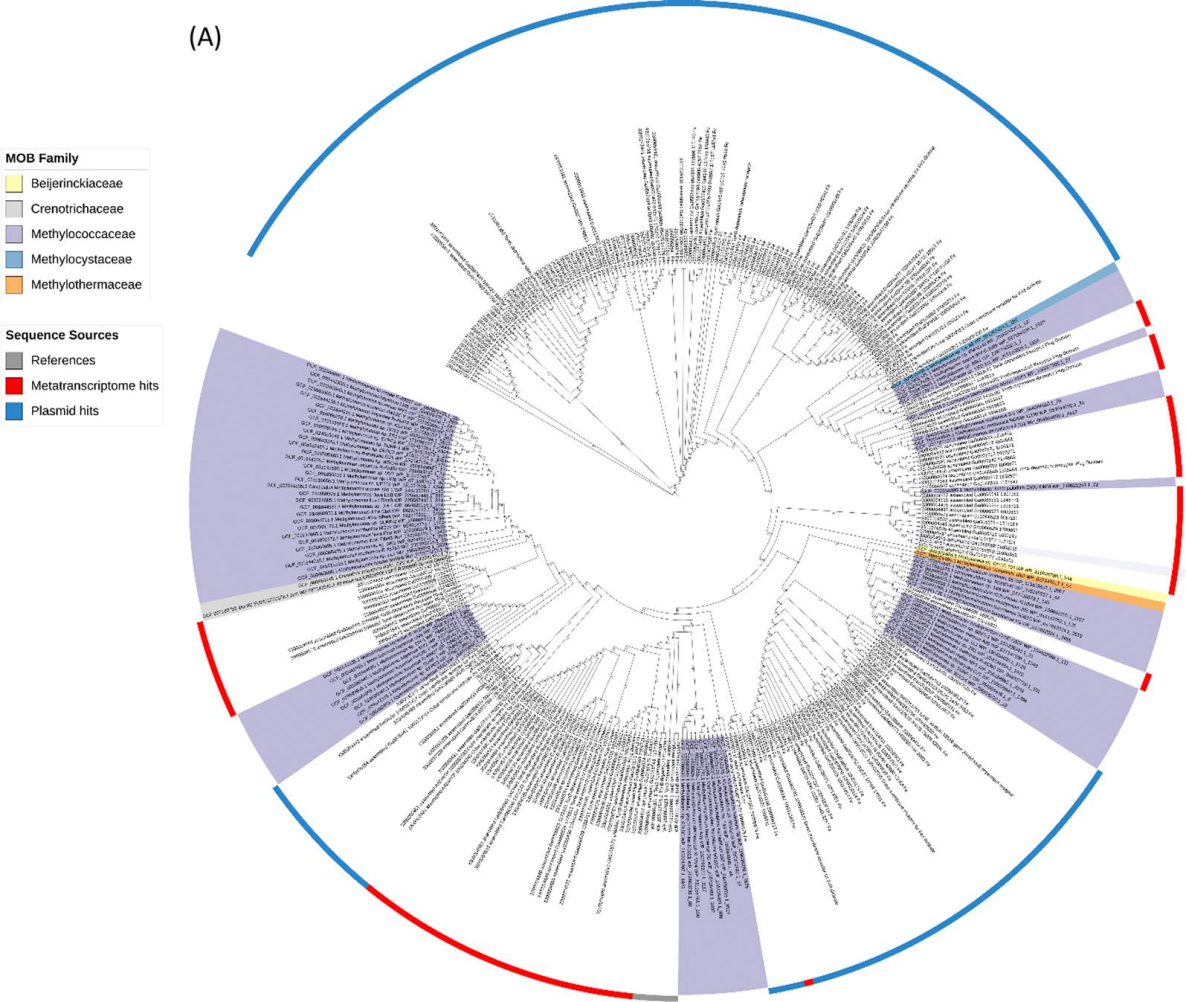

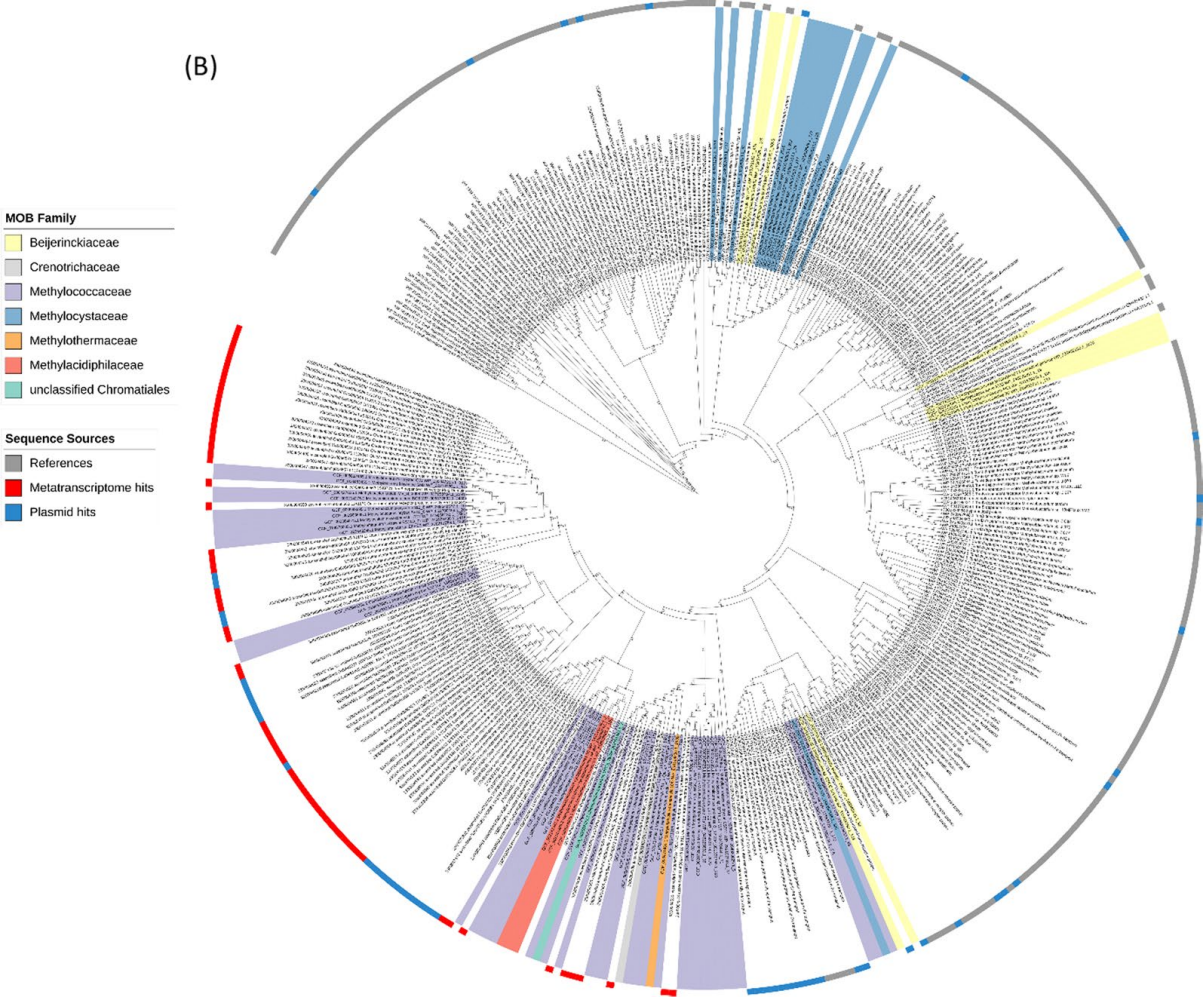
Phylogenetic distribution of LanA and LutH-like sequences in MOB genomes, plasmids, and metatranscriptomes. Circular phylogenetic trees showing all retrieved **A** LanA and **B** LutH-like sequences from MOB genomes (coloured ranges), plasmids (blue strips), lake sediment metatranscriptomes (red strips), and reference sequences (grey strips). The trees were annotated using iTOL; label colour ranges indicate MOB families. Bootstrap support values (based on 1,000 replicates) are shown on nodes to one decimal place

We also analysed the TonB-dependent receptors **LanA** and **LutH,** first identified in *Methylotuvimicrobium buryatense* 5GB1C and *M. extorquens* AM1, respectively. Both genes were found to be crucial in controlling the lanthanide switch in these organisms, particularly in the lanthanide uptake system [17, 20]. LanA from *M. buryatense* 5GB1C was the first lanthanum receptor identified in a methanotroph. Recently, Shiina and colleagues [18] identified a *lutH*-like gene in *Methylosinus trichosporium* OB3b. Our work revealed that LanA is widely distributed in MOBs, predominantly within the *Methylococcaceae* family. We also detected two LanA genes from the family *Crenotrichaceae* and one each from *Methylothermaceae*, *Methylocystaceae*, and *Beijerinckiaceae*. Interestingly, this gene was also identified in metatranscriptomes related to *Methylobacter*, *Methylomonas*, and *Methylotenera*; however, while several LanA were found in plasmids, none related to MOB was found (Fig. 6a). Similarly, the *lutH*-like gene was found in all the MOB families where *lanA* was detected, including the *Methylococcaceae, Crenotrichaceae, Methylothermaceae, Methylocystaceae, Beijerinckiaceae,* as well as in *Methylacidiphilaceae* and unclassified *Chromatiales.* LutH was also found in metatranscriptomes and plasmids (Fig. 6b). Interestingly, the evolutionary relationships of *lanA* and *lutH*-like genes do not align with the known phylogeny of their host organisms. This mismatch, known as phylogenetic incongruence, suggests that some of these genes may have been acquired through horizontal gene transfer, especially given their presence on plasmids from unrelated taxa.

This study focuses on the distribution of lanthanide-associated genes across a curated dataset of verified aerobic methanotroph genomes. While informative, the dataset reflects the current availability of high-quality isolate genomes and is constrained by the taxonomic representation within publicly curated databases. As such, the observed patterns, such as apparent lineage-specific gene presence, should not be interpreted as representative of all methanotrophic diversity. Statistical inference was not applied here, as the dataset was assembled primarily for gene discovery and contextual analysis in well-characterised MOB genomes rather than for broad phylogenetic generalisation. Future studies would benefit from expanding this approach to include uncultivated and environmentally abundant methanotrophs. Leveraging comprehensive resources such as the Genome Taxonomy Database (GTDB) could enhance the phylogenetic breadth and ecological representation of MOB, enabling more profound insights into the distribution and evolution of lanthanide-associated traits across diverse microbial lineages.

### Impact of cerium and mixed lanthanides on methane oxidation and protein expression in *M. trichosporium* OB3b

**Fig. 7:**
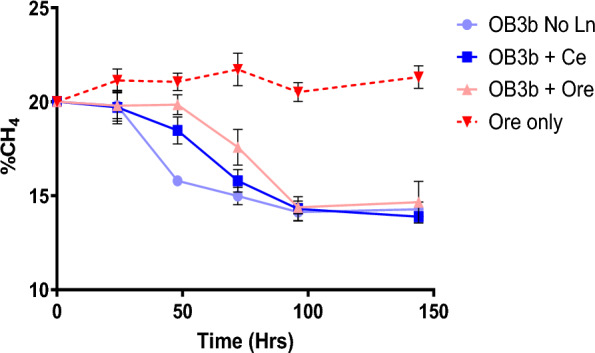
Methane oxidation by *M. trichosporium* OB3b in the presence and absence of lanthanides (cerium and mixed lanthanides). Methane concentrations (% v/v) were monitored over 6 days in triplicate batch incubations. Data represent mean values; error bars indicate standard deviation (n = 3)

Rare earth elements (REE) are naturally present in the environment, where they predominantly occur as insoluble oxides. To investigate how an aerobic MOB responds to this environmentally relevant form compared to the commonly used soluble laboratory form (CeCl_3_), we assessed methane oxidation of *M. trichosporium* OB3b under three conditions: no added lanthanides (No Ln), with cerium (Ce), and with a lanthanide-rich ore. These treatments revealed distinct patterns in bacterial methane oxidation activity in response to the presence and absence of different Ln sources (Fig. 7). The longest lag phase was observed in the ore-treated culture, followed by the Ce-treated culture, while the No Ln control exhibited the shortest lag phase. These differences suggest that *M. trichosporium* OB3b requires additional time to adjust its regulatory and metabolic pathways in the presence of lanthanides. In particular, the Ore treatment likely presents additional physiological challenges due to its heterogeneous, relatively insoluble composition, which may require greater cellular effort for metal solubilisation, uptake, and coping with potential toxicity. In contrast, the Ce treatment may allow faster adaptation as Ce^3+^ is a single, soluble cofactor, reducing the complexity of metal handling.

Although we attempted to track biomass accumulation using optical density measurements, this was not feasible for the ore-treated cultures due to interference from suspended fine particles. Therefore, we used methane consumption as a proxy for metabolic activity and growth. As previously reported, the presence or absence of lanthanides can influence the growth and methane oxidation rates of methanotrophs, including *M. trichosporium* OB3b and related species [4, 61, 62]. Despite longer lag phases in the Ln-treated cultures, the average methane oxidation rate calculated between 24 and 96 h was comparable across all treatments, approximately 0.08% CH_4_ hr^−1^. Specifically, the highest methane oxidation rate was observed in the control treatment (0.17% CH₄ hr⁻^1^ between 24 and 48 h), while the OB3b + Ce and OB3b + Ore treatments exhibited slightly lower rates (0.11% CH₄ hr⁻^1^ between 48 and 72 h). These results suggest that although methane oxidation may be delayed in the presence of lanthanides, *M. trichosporium* OB3b can adapt to their presence and ultimately achieve similar methane oxidation capacities as cultures grown without Ln. This implies that environmentally or geologically relevant lanthanide sources, when present in sufficient quantities, can support methane oxidation by MOBs following an initial period of acclimation. Future studies should, however, consider lanthanide concentrations that reflect typical environmental levels to enhance ecological relevance.

It is worth noting that, while we did not determine gene expression during the lag phase, our endpoint proteomic analysis revealed marked differences in protein expression between treatments, particularly among lanthanide transporters and enzymes (Table 1). These changes support the hypothesis that the lag phase involves a regulatory and metabolic shift required for efficient lanthanide acquisition and utilisation, supporting the view that physiological plasticity in bacteria, especially in protein synthesis and metabolic adaptation, may contribute to the lag phase [63–65].

**Table 1:** Differential expression of MDH and lanthanide-associated proteins in *M. trichosporium* OB3b in response to cerium (Ce) and mixed lanthanide ore (Ore) treatments. Log₂ fold changes (log₂FC) are shown for each treatment relative to the control (OB3b with no lanthanide). Genes are grouped according to whether they were upregulated or downregulated in both treatments. LanM was not detected as differentially expressed in either condition

Protein/locus number	Putative Function	log₂FC (Ce)	log₂FC (Ore)
*Upregulated in cerium and ore treatments compared with no lanthanide*			
XoxF3 (MettrDRAFT_1695)	Methanol dehydrogenase large subunit (Ln^3^⁺-dependent, XoxF3-type)	7.66	5.37
XoxF5 (MettrDRAFT_1984)	Methanol dehydrogenase large subunit (Ln^3^⁺-dependent, XoxF5-type)	2.65	4.52
XoxJ (MettrDRAFT_1694)	Cytochrome c, putative electron acceptor for XoxF-type MDH	2.16	0.83
TonB-dependent siderophore receptor (MettrDRAFT_4452)	Outer membrane receptor for siderophores/lanthanophores, may mediate Ln uptake	2.44	1.68
ABC transporter (MettrDRAFT_4451)	Cyclic peptide export ABC transporter; component of possible lanthanophore or metal transport system	4.77	1.68
*Downregulated in cerium and ore treatments compared with no lanthanide*			
TonB-dependent receptor (LutH-like) (MettrDRAFT_0198)	Lanthanide/iron complex outer membrane receptor protein. Mediate Ln uptake	− 3.16	− 1.92
MxaF (MettrDRAFT_1978)	Methanol dehydrogenase large subunit (Ca^2^⁺-dependent, MxaFI-type)	− 3.63	− 2.32
MxaA (MettrDRAFT_1972)	Methanol dehydrogenase small subunit (Ca^2^⁺-dependent, MxaFI-type)	− 3.03	− 4.11
MxaJ (MettrDRAFT_1977)	Cytochrome cL; electron acceptor for MxaFI-type MDH	− 5.7	− 4.4
MxaC (MettrDRAFT_1971)	Mxa methyltransferase, function in MDH maturation/assembly	− 1.33	− 2.05
MxaD (MettrDRAFT_1968)	MxaD protein, involved in MDH assembly or regulation	− 5.3	− 5.84
MxaK (MettrDRAFT_1970)	MxaK, methanol utilization (possible kinase/regulator)	− 2.87	− 3.49
MxaL (MettrDRAFT_1969)	MxaL, periplasmic protein, function in MDH maturation/assembly	0	− 2.08
*Not differentially expressed*			
LanM (MettrDRAFT_1017)	Lanmodulin; high-affinity lanthanide-binding periplasmic protein	ND	ND

The data show the log2 fold-change (FC) of proteins upregulated or downregulated in both the cerium and ore treatments. Significance is based on FDR-adjusted p-values. ND: Not detected

Proteomic analysis revealed significant changes in protein expression in both the Ce and Ore treatments compared to the control. A total of 1,414 proteins were detected, with 60 and 724 proteins showing significant differential expression in the Ce and Ore treatments, respectively, compared to the no lanthanide control (Table S6). The 12-fold greater number of differentially expressed proteins in the Ore treatment compared to the Ce-only treatment is likely attributable to the complex nature and composition of the ore. The response of *M. trichosporium* OB3b to the ore is likely not only a response to the provision of lanthanides in a more complex and less bioavailable form but also a response to other metals in the ore, resulting in the induction of various proteins, including those associated with stress response and chemotaxis. This response is necessary for the organism to adapt and thrive in its environment. In the Ce-only treatment, among the 60 significantly expressed proteins, 33 were upregulated, and 27 were downregulated. In the Ore treatment, 144 proteins were upregulated, while 580 were downregulated.

Our genome analysis of *M. trichosporium* OB3b revealed the presence of genes encoding *mxaF, xoxF3*, *xoxF5, lanM*, and *lutH*-like TonB-dependent receptor. Proteomic analysis confirmed the presence of the products of all these genes except LanM in both the Ce and Ore treatments (Table 1). For LanM, only a single peptide fragment was observed in the control and Ce samples; however, this fell below the MaxQuant threshold required for confident identification/quantification. Furthermore, the lanthanide-dependent enzymes XoxF3 and XoxF5 were upregulated, while the calcium-dependent MxaF and the LutH-like protein were downregulated under both conditions (Table 1). The gene encoding MxaF is in a 12-gene cluster implicated in calcium-dependent MDH activity, eight of which (including *mxaF* and *mxaJ*) were downregulated under both conditions. In contrast, a homologous protein to MxaJ, XoxJ, which is encoded adjacent to *xoxF3*, was upregulated in both Ce- and ore-treatment. The inverse expression/regulation of MxaF and XoxF and their associated genes in response to lanthanide bioavailability strongly suggests that XoxF lanthanide-binding proteins play a critical role in the utilisation of lanthanides in *M. trichosporium* OB3b. The inverse regulation of MxaF and XoxF due to lanthanide availability has been previously demonstrated [11].

Interestingly, LanM, which is known to respond to picomolar concentrations of lanthanides in obligate methylotrophs, was not expressed in the presence of Ce or Ore treatments. This suggests that while *M. trichosporium* OB3b possesses the *lanM* gene, it may not be the primary gene responsible for lanthanide acquisition and trafficking in this bacterium under the growth conditions we have tested. Recent studies by Shiina et al. (2023) demonstrated that the LutH-like TonB-dependent receptor (CQW49_RS02145) is required for the expression of XoxF in the presence of cerium in a laboratory-adapted strain of *M. trichosporium* OB3b [18]. These findings imply a crucial role for this gene in lanthanide uptake across the outer membrane. However, in our study, the LutH-like gene (MettrDRAFT_0198) was downregulated in both the Ce and Ore treatments. This observation aligns with findings by Gu and Semrau [42], who reported downregulation of the same gene (referred to as ADVE02_v2_10208) in the presence of cerium and the absence of copper. Such downregulation of the LutH-like gene may be due to the high concentration of Ce, as metal ion uptake systems are typically downregulated when intracellular metal levels reach sufficient concentrations to maintain homeostasis [66, 67]. Our findings are consistent with MettrDRAFT_0198 LutH-like as a component of a primary route for lanthanide uptake and trafficking in *M. trichosporium* OB3b. Its downregulation under elevated Ce conditions supports a model in which the bacterium modulates uptake systems to avoid excess metal accumulation, thus maintaining intracellular metal homeostasis. Although copper has been shown to modulate the lanthanide switch and influence expression of MDH in *M. trichosporium* OB3b [42, 44], no copper was added to either the cerium or Ore treatments in our study, and no substantial background copper was detected. Therefore, the observed expression patterns, particularly the upregulation of XoxF and the downregulation of MxaF and LutH-like proteins, can be attributed primarily to lanthanide exposure. These results support a model in which lanthanides alone, without copper interference, are sufficient to drive the switch in MDH expression and associated metal uptake pathways under the tested conditions.

The large number of proteins that are differently affected by the two lanthanide sources, including some 48 transport-associated proteins, along with 19 putative molecular chaperones, may indicate proteins that are necessary for liberating lanthanides from geological sources and growing in the presence of the stresses, lanthanide-related and others, that such materials present.

Among the proteins significantly upregulated in both Ce and Ore treatments, we identified a TonB-dependent siderophore receptor (MettrDRAFT_4452) and an adjacent cyclic peptide export ABC transporter (MettrDRAFT_4451). This co-upregulation suggests a coordinated role in lanthanide acquisition, potentially through a lanthanophore-mediated mechanism similar to bacterial siderophore-iron uptake systems. Recent work by Juma et al. (2022) demonstrated that *Methylobacterium aquaticum* strain 22A utilises a siderophore to solubilise lanthanides, facilitating their uptake through a TonB-dependent receptor [68]. This raises the possibility that *M. trichosporium* OB3b employs a similar strategy, where the cyclic peptide export ABC transporter secretes metal-chelating peptides that enhance lanthanide solubilisation, while the TonB-dependent receptor mediates uptake of the resulting lanthanide-peptide complex [68]. This system may represent an important alternative lanthanide-scavenging strategy in *M. trichosporium* OB3b, distinct from the previously characterised LutH-like transport pathway.

## Conclusion

This study presents a focused genomic and proteomic investigation of the lanthanome in aerobic MOB, integrating isolate genomes, plasmids, environmental transcriptomes, and laboratory proteomics. We show that lanthanoenzymes such as XoxF are distributed across all major phyla, with *xoxF5* being the most prevalent, particularly among Proteobacteria, where multiple gene copies often occur. In contrast, lanthanide transporters show a more restricted and clade-specific distribution. LanM was detected only in alphaproteobacterial MOBs, while *lanA* and *LanPepSY* were largely associated with gammaproteobacterial MOBs, primarily within the *Methylococcaceae*. The *lutH*-like gene showed the widest phylogenetic distribution. These patterns suggest that different methanotrophs employ distinct molecular systems for acquiring, trafficking, and utilising lanthanides, with no universal transport mechanism across the group. In addition to chromosomal genes, we detected lanthanide-associated genes on MOB and non-MOB plasmids, suggesting potential roles in horizontal gene transfer and rapid environmental adaptation. The detection of lanthanome transcripts in environmental metatranscriptomes further supports their ecological activity in natural systems.

Experimental analysis of *M. trichosporium* OB3b exposed to soluble cerium and ore-derived lanthanides revealed distinct condition-specific responses in methane oxidation and protein expression. While lag phases differed, methane oxidation rates became comparable following acclimation. Proteomic analysis showed broader changes under Ore treatment, possibly due to its complex mineral composition. The “lanthanide switch” was evident, with upregulation of *xoxF3* and *xoxF5* and downregulation of *mxaF* and its accessory genes. Interestingly, although LanM is encoded in the genome, but it was not detected at the protein level in any treatment. The *lutH*-like gene was consistently downregulated under both treatments, possibly reflecting regulatory control to maintain intracellular metal homeostasis after uptake.

Together, our findings expand current knowledge of the distribution, regulation, and potential mobility of Ln-related systems in MOBs. The lanthanome gene inventory, combined with proteomic and metatranscriptomic evidence, provides a valuable resource for future functional and ecological studies. In particular, the identification of diverse Ln-binding and transport genes across genomes and plasmids highlights candidate targets for biotechnological development. For instance, engineered strains expressing specific Ln-binding or transport proteins could be used to selectively bind, solubilise, and extract lanthanides from low-grade ores, mine waste, or electronic waste streams. Moreover, understanding the regulation of these pathways may support the development of biosensors and biocatalysts for rare earth element recovery, contributing to sustainable bioprocessing technologies.

## Supplementary Information

Additional file 1.

Additional file 2.

Additional file 3.

Additional file 4.

## Data Availability

The proteomics data have been deposited in the ProteomeXchange Consortium via the PRIDE partner repository with the dataset identifier PXD063434.

## Abbreviations

Ce: Cerium
Ln: Lanthanides
MDH: Methanol dehydrogenase
MOB: Methane oxidising bacteria
LanM: Lanmodulin
LanP: LanPepSY
MAGs: Metagenome assembled genomes
HGT: Horizontal gene transfer

## References

[1] Fitriyanto NA, Fushimi M, Matsunaga M, Pertiwiningrum A, Iwama T, Kawai K. Molecular structure and gene analysis of Ce^3+^-induced methanol dehydrogenase of *Bradyrhizobium* sp. MAFF211645. J Biosci Bioeng. 2011;111:613–7. 21334970 10.1016/j.jbiosc.2011.01.015

[2] Hibi Y, Asai K, Arafuka H, Hamajima M, Iwama T, Kawai K. Molecular structure of La^3+^-induced methanol dehydrogenase-like protein in *Methylobacterium radiotolerans*. J Biosci Bioeng. 2011;111:547–9. 21256798 10.1016/j.jbiosc.2010.12.017

[3] Nakagawa T, Mitsui R, Tani A, Sasa K, Tashiro S, Iwama T, et al. A catalytic role of XoxF1 as La^3+^-dependent methanol dehydrogenase in *Methylobacterium extorquens* strain AM1. PLoS ONE. 2012;7:e50480. 23209751 10.1371/journal.pone.0050480 PMC3507691

[4] Pol A, Barends TRM, Dietl A, Khadem AF, Eygensteyn J, Jetten MSM, et al. Rare earth metals are essential for methanotrophic life in volcanic mudpots. Environ Microbiol. 2014;16:255–64. 24034209 10.1111/1462-2920.12249

[5] Karthikeyan OP, Smith TJ, Dandare SU, Parwin KS, Singh H, Loh HX, et al. Metal(loid) speciation and transformation by aerobic methanotrophs. Microbiome. 2021;9:1–18. 34229757 10.1186/s40168-021-01112-y PMC8262016

[6] Chistoserdova L. Modularity of methylotrophy, revisited. Environ Microbiol. 2011;13:2603–22. 21443740 10.1111/j.1462-2920.2011.02464.x

[7] Taubert M, Grob C, Howat AM, Burns OJ, Dixon JL, Chen Y, et al. XoxF encoding an alternative methanol dehydrogenase is widespread in coastal marine environments. Environ Microbiol. 2015;17:3937–48. 25943904 10.1111/1462-2920.12896

[8] Kalyuzhnaya MG, Puri AW, Lidstrom ME. Metabolic engineering in methanotrophic bacteria. Metab Eng. 2015;29:142–52. 25825038 10.1016/j.ymben.2015.03.010

[9] Fei Q, Guarnieri MT, Tao L, Laurens LML, Dowe N, Pienkos PT. Bioconversion of natural gas to liquid fuel: opportunities and challenges. Biotechnol Adv. 2014;32:596–614. 24726715 10.1016/j.biotechadv.2014.03.011

[10] Crombie AT. The effect of lanthanum on growth and gene expression in a facultative methanotroph. Environ Microbiol. 2022;24:596–613. 34320271 10.1111/1462-2920.15685 PMC9291206

[11] Masuda S, Suzuki Y, Fujitani Y, Mitsui R, Nakagawa T, Shintani M, et al. Lanthanide-dependent regulation of methylotrophy in *Methylobacterium aquaticum* strain 22A. mSphere. 2018;3:e00462–17. 29404411 10.1128/mSphere.00462-17 PMC5784242

[12] Huang J, Yu Z, Chistoserdova L. Lanthanide-dependent methanol dehydrogenases of XoxF4 and XoxF5 clades are differentially distributed among methylotrophic bacteria and they reveal different biochemical properties. Front Microbiol. 2018;9:376220. 10.3389/fmicb.2018.01366 PMC6028718 29997591

[13] Keltjens JT, Pol A, Reimann J, den Op Camp HJM. PQQ-dependent methanol dehydrogenases: Rare-earth elements make a difference. Appl Microbiol Biotechnol. 2014;98:6163–83. 24816778 10.1007/s00253-014-5766-8

[14] Wehrmann M, Billard P, Martin-Meriadec A, Zegeye A, Klebensberger J. Functional role of lanthanides in enzymatic activity and transcriptional regulation of pyrroloquinoline quinone-dependent alcohol dehydrogenases in *Pseudomonas putida* KT2440. MBio. 2017. 10.1128/mBio.00570-17. 28655819 10.1128/mBio.00570-17 PMC5487730

[15] Voutsinos MY, Banfield JF, McClelland HLO. Extensive and diverse lanthanide-dependent metabolism in the ocean. ISME J. 2025. 10.1093/ismejo/wraf057. 40121542 10.1093/ismejo/wraf057 PMC11996626

[16] Cotruvo JA, Featherston ER, Mattocks JA, Ho JV, Laremore TN. Lanmodulin: a highly selective lanthanide-binding protein from a lanthanide-utilizing bacterium. J Am Chem Soc. 2018;140:15056–61. 30351021 10.1021/jacs.8b09842

[17] Groom JD, Ford SM, Pesesky MW, Lidstrom ME. A mutagenic screen identifies a TonB-dependent receptor required for the lanthanide metal switch in the type i methanotroph ‘methylotuvimicrobium buryatense’ 5GB1C. J Bacteriol. 2019;201(15):10–128. 10.1128/JB.00120-19 PMC6620403 31085692

[18] Shiina W, Ito H, Kamachi T. Identification of a TonB-Dependent Receptor Involved in Lanthanide Switch by the Characterization of Laboratory-Adapted Methylosinus trichosporium OB3b. Appl Environ Microbiol. 2023;89:e01413-e1422. 36645275 10.1128/aem.01413-22 PMC9888264

[19] Ochsner AM, Hemmerle L, Vonderach T, Nüssli R, Bortfeld-Miller M, Hattendorf B, et al. Use of rare-earth elements in the phyllosphere colonizer Methylobacterium extorquens PA1. Mol Microbiol. 2019;111:1152–66. 30653750 10.1111/mmi.14208 PMC6850437

[20] Roszczenko-Jasińska P, Vu HN, Subuyuj GA, Crisostomo RV, Cai J, Lien NF, et al. Gene products and processes contributing to lanthanide homeostasis and methanol metabolism in Methylorubrum extorquens AM1. Sci Rep. 2020;10:1–15. 32728125 10.1038/s41598-020-69401-4 PMC7391723

[21] Hemmann JL, Keller P, Hemmerle L, Vonderach T, Ochsner AM, Bortfeld-Miller M, et al. Lanpepsy is a novel lanthanide-binding protein involved in the lanthanide response of the obligate methylotroph *Methylobacillus flagellatus*. J Biol Chem. 2023. 10.1016/j.jbc.2023.102940. 36702252 10.1016/j.jbc.2023.102940 PMC9988556

[22] Edwards CR, Onstott TC, Miller JM, Wiggins JB, Wang W, Lee CK, et al. Draft genome sequence of uncultured upland soil cluster gammaproteobacteria gives molecular insights into high-affinity methanotrophy. Genome Announc. 2017;5(17):10–28. 10.1128/genomeA.00047-17 PMC5408097 28450499

[23] Pratscher J, Vollmers J, Wiegand S, Dumont MG, Kaster AK. Unravelling the identity, metabolic potential and global biogeography of the atmospheric methane-oxidizing upland soil cluster α. Environ Microbiol. 2018;20:1016–29. 29314604 10.1111/1462-2920.14036 PMC6849597

[24] Tveit AT, Hestnes AG, Robinson SL, Schintlmeister A, Dedysh SN, Jehmlich N, et al. Widespread soil bacterium that oxidizes atmospheric methane. Proc Natl Acad Sci U S A. 2019;116:8515–24. 30962365 10.1073/pnas.1817812116 PMC6486757

[25] van Spanning RJM, Guan Q, Melkonian C, Gallant J, Polerecky L, Flot JF, et al. Methanotrophy by a *Mycobacterium* species that dominates a cave microbial ecosystem. Nat Microbiol. 2022;7:12. 2022;7:2089–100. 36329197 10.1038/s41564-022-01252-3

[26] Edgar RC. Muscle: a multiple sequence alignment method with reduced time and space complexity. BMC Bioinformatics. 2004;5:1–19. 15318951 10.1186/1471-2105-5-113 PMC517706

[27] Capella-Gutiérrez S, Silla-Martínez JM, Gabaldón T. TrimAl: a tool for automated alignment trimming in large-scale phylogenetic analyses. Bioinformatics. 2009;25:1972–3. 19505945 10.1093/bioinformatics/btp348 PMC2712344

[28] Price MN, Dehal PS, Arkin AP. Fasttree 2 – approximately maximum-likelihood trees for large alignments. PLoS ONE. 2010;5:e9490. 20224823 10.1371/journal.pone.0009490 PMC2835736

[29] Eddy SR. Accelerated profile HMM searches. PLoS Comput Biol. 2011;7:e1002195. 22039361 10.1371/journal.pcbi.1002195 PMC3197634

[30] Hyatt D, Locascio PF, Hauser LJ, Uberbacher EC. Gene and translation initiation site prediction in metagenomic sequences. Bioinformatics. 2012;28:2223–30. 22796954 10.1093/bioinformatics/bts429

[31] Lee MD. Gtotree: a user-friendly workflow for phylogenomics. Bioinformatics. 2019;35:4162–4. 30865266 10.1093/bioinformatics/btz188 PMC6792077

[32] Camargo AP, Call L, Roux S, Nayfach S, Huntemann M, Palaniappan K, et al. IMG/PR: a database of plasmids from genomes and metagenomes with rich annotations and metadata. Nucleic Acids Res. 2024;52:D164–73. 37930866 10.1093/nar/gkad964 PMC10767988

[33] Zheng Y, Wang H, Yu Z, Haroon F, Hernández ME, Chistoserdova L. Metagenomic insight into environmentally challenged methane-fed microbial communities. Microorganisms. 2020;8:1614. 33092280 10.3390/microorganisms8101614 PMC7589939

[34] Finn RD, Clements J, Eddy SR. HMMER web server: interactive sequence similarity searching. Nucleic Acids Res. 2011;39:W29-37. 21593126 10.1093/nar/gkr367 PMC3125773

[35] Aramaki T, Blanc-Mathieu R, Endo H, Ohkubo K, Kanehisa M, Goto S, et al. KofamKOALA: KEGG ortholog assignment based on profile HMM and adaptive score threshold. Bioinformatics. 2020;36:2251–2. 31742321 10.1093/bioinformatics/btz859 PMC7141845

[36] Shen W, Le S, Li Y, Hu F. SeqKit: a cross-platform and ultrafast toolkit for FASTA/Q file manipulation. PLoS ONE. 2016;11:e0163962. 27706213 10.1371/journal.pone.0163962 PMC5051824

[37] Sievers F, Higgins DG. Clustal omega for making accurate alignments of many protein sequences. Protein Sci. 2018;27:135–45. 28884485 10.1002/pro.3290 PMC5734385

[38] Scanlan J, Guillonneau R, Cunningham MR, Najmin S, Mausz MA, Murphy A, et al. The proteobacterial methanotroph *Methylosinus trichosporium* OB3b remodels membrane lipids in response to phosphate limitation. MBio. 2022. 10.1128/mbio.00247-22. 35575546 10.1128/mbio.00247-22 PMC9239053

[39] Fu L, Niu B, Zhu Z, Wu S, Li W. CD-HIT: accelerated for clustering the next-generation sequencing data. Bioinformatics. 2012;28:3150–2. 23060610 10.1093/bioinformatics/bts565 PMC3516142

[40] Letunic I, Bork P. Interactive tree of life (iTOL) v5: an online tool for phylogenetic tree display and annotation. Nucleic Acids Res. 2021;49:W293–6. 33885785 10.1093/nar/gkab301 PMC8265157

[41] Smith TJ, Murrell JC. Mutagenesis of soluble methane monooxygenase. Methods Enzymol. 2011;495:135–47. 21419919 10.1016/B978-0-12-386905-0.00009-7

[42] Gu W, Semrau JD. Copper and cerium-regulated gene expression in *Methylosinus trichosporium* OB3b. Appl Microbiol Biotechnol. 2017;101:8499–516. 29032471 10.1007/s00253-017-8572-2

[43] Farhan Ul Haque M, Gu W, DiSpirito AA, Semrau JD. Marker exchange mutagenesis of mxaF, encoding the large subunit of the Mxa methanol dehydrogenase, in *Methylosinus trichosporium* OB3b. Appl Environ Microbiol. 2016;82:1549–55. 10.1128/AEM.03615-15 PMC4771321 26712545

[44] Ul Haque MF, Kalidass B, Bandow N, Turpin EA, DiSpirito AA, Semrau JD. Cerium regulates expression of alternative methanol dehydrogenases in Methylosinus trichosporium OB3b. Appl Environ Microbiol. 2015;81:7546–52. 26296730 10.1128/AEM.02542-15 PMC4592857

[45] Meier F, Brunner AD, Koch S, Koch H, Lubeck M, Krause M, et al. Online parallel accumulation-serial fragmentation (PASEF) with a novel trapped ion mobility mass spectrometer. Mol Cell Proteomics. 2018;17:2534–45. 30385480 10.1074/mcp.TIR118.000900 PMC6283298

[46] Cox J, Mann M. Maxquant enables high peptide identification rates, individualized p.p.b.-range mass accuracies and proteome-wide protein quantification. Nat Biotechnol. 2008;26:1367–72. 19029910 10.1038/nbt.1511

[47] Perez-Riverol Y, Bandla C, Kundu DJ, Kamatchinathan S, Bai J, Hewapathirana S, et al. The PRIDE database at 20 years: 2025 update. Nucleic Acids Res. 2025;53:D543–53. 39494541 10.1093/nar/gkae1011 PMC11701690

[48] Samanta D, Rauniyar S, Saxena P, Sani RK. From genome to evolution: investigating type II methylotrophs using a pangenomic analysis. mSystems. 2024. 10.1128/msystems.00248-24. 38695578 10.1128/msystems.00248-24 PMC11237726

[49] Dedysh SN, Knief C, Dunfield PF. *Methylocella* species are facultatively methanotrophic. J Bacteriol. 2005;187:4665. 15968078 10.1128/JB.187.13.4665-4670.2005 PMC1151763

[50] Huang J, Yu Z, Groom J, Cheng JF, Tarver A, Yoshikuni Y, et al. Rare earth element alcohol dehydrogenases widely occur among globally distributed, numerically abundant and environmentally important microbes. ISME J. 2019;13:2005–17. 30952993 10.1038/s41396-019-0414-z PMC6775964

[51] Ramachandran A, Walsh DA. Investigation of XoxF methanol dehydrogenases reveals new methylotrophic bacteria in pelagic marine and freshwater ecosystems. FEMS Microbiol Ecol. 2015. 10.1093/femsec/fiv105. 26324853 10.1093/femsec/fiv105

[52] Lynch M, Conery JS. The evolutionary fate and consequences of duplicate genes. Science. 1979;2000(290):1151–5. 10.1126/science.290.5494.1151 11073452

[53] Macey MC, Pratscher J, Crombie AT, Murrell JC. Impact of plants on the diversity and activity of methylotrophs in soil. Microbiome. 2020;8:1–17. 32156318 10.1186/s40168-020-00801-4 PMC7065363

[54] van Teeseling MCF, Pol A, Harhangi HR, van der Zwart S, Jetten MSM, den Op Camp HJM, et al. Expanding the verrucomicrobial methanotrophic world: description of three novel species of Methylacidimicrobium gen nov. Appl Environ Microbiol. 2014;80:6782–91. 25172849 10.1128/AEM.01838-14 PMC4249049

[55] Osborne CD, Haritos VS. Horizontal gene transfer of three co-inherited methane monooxygenase systems gave rise to methanotrophy in the Proteobacteria. Mol Phylogenet Evol. 2018;129:171–81. 30149053 10.1016/j.ympev.2018.08.010

[56] Chistoserdova L. Methylotrophy in a lake: from metagenomics to single-organism physiology. Appl Environ Microbiol. 2011;77:4705. 21622781 10.1128/AEM.00314-11 PMC3147377

[57] Islam MM, Le T, Daggumati SR, Saha R. Investigation of microbial community interactions between Lake Washington methanotrophs using genome-scale metabolic modeling. PeerJ. 2020;2020:e9464. 10.7717/peerj.9464 PMC7333651 32655999

[58] Yu Z, Groom J, Zheng Y, Chistoserdova L, Huang J. Synthetic methane-consuming communities from a natural lake sediment. MBio. 2019. 10.1128/mBio.01072-19. 31337718 10.1128/mBio.01072-19 PMC6650549

[59] Mattocks JA, Jung JJ, Lin CY, Dong Z, Yennawar NH, Featherston ER, et al. Enhanced rare-earth separation with a metal-sensitive lanmodulin dimer. Nature. 2023;618:87–93. 37259003 10.1038/s41586-023-05945-5 PMC10232371

[60] Yang W, Wu K, Chen H, Huang J, Yu Z. Emerging role of rare earth elements in biomolecular functions. ISME J. 2025;19:241. 10.1093/ismejo/wrae241 PMC11845868 39657633

[61] Crombie AT, Murrell JC. Trace-gas metabolic versatility of the facultative methanotroph *Methylocella silvestris*. Nature. 2014;510:7503. 2014;510:148–51. 10.1038/nature13192 24776799

[62] Vu HN, Subuyuj GA, Vijayakumar S, Good NM, Martinez-Gomez NC, Skovran E. Lanthanide-dependent regulation of methanol oxidation systems in *Methylobacterium extorquens* AM1 and their contribution to methanol growth. J Bacteriol. 2016;198:1250. 26833413 10.1128/JB.00937-15 PMC4859578

[63] Bertranda RL. Lag phase is a dynamic, organized, adaptive, and evolvable period that prepares bacteria for cell division. J Bacteriol. 2019;201:e00697-e718. 30642990 10.1128/JB.00697-18 PMC6416914

[64] Hamill PG, Stevenson A, McMullan PE, Williams JP, Lewis ADR, Sudharsan S, et al. Microbial lag phase can be indicative of, or independent from, cellular stress. Sci Rep. 2020;10:1–20. 32246056 10.1038/s41598-020-62552-4 PMC7125082

[65] Rolfe MD, Rice CJ, Lucchini S, Pin C, Thompson A, Cameron ADS, et al. Lag phase is a distinct growth phase that prepares bacteria for exponential growth and involves transient metal accumulation. J Bacteriol. 2012;194:686. 22139505 10.1128/JB.06112-11 PMC3264077

[66] Chandrangsu P, Rensing C, Helmann JD. Metal homeostasis and resistance in bacteria. Nat Rev Microbiol. 2017;15:338–50. 28344348 10.1038/nrmicro.2017.15 PMC5963929

[67] Baksh KA, Zamble DB. Allosteric control of metal-responsive transcriptional regulators in bacteria. J Biol Chem. 2020;295:1673–84. 31857375 10.1074/jbc.REV119.011444 PMC7008368

[68] Juma PO, Fujitani Y, Alessa O, Oyama T, Yurimoto H, Sakai Y, et al. Siderophore for lanthanide and iron uptake for methylotrophy and plant growth promotion in *Methylobacterium aquaticum* strain 22A. Front Microbiol. 2022;13:921635. 35875576 10.3389/fmicb.2022.921635 PMC9301485

