# Supplementary material for: Diversity and distribution of the lanthanome in aerobic methane-oxidising bacteria
Source: Environ Microbiome. 2025 Sep 29;20:120. doi: 10.1186/s40793-025-00776-5 (PMC12482762; doi:10.1186/s40793-025-00776-5)
Supplement: Supplementary file 1 — Additional file 1. [file 40793_2025_776_MOESM1_ESM.docx]

**Supplementary Information**

**Diversity and distribution of the lanthanome in aerobic methane-oxidising bacteria**

Shamsudeen Umar Dandare^1,2^, Alexander Allenby^1^, Eleonora Silvano^3,4^, Peter Nockemann^5^, Yin Chen^4^, Thomas J Smith^6^, Deepak Kumaresan^1,4^

Contents:

- Tables S1-S6
- Figures S1-S5

**Supporting Tables**

**Table S1:** MOB custom database including complete names, genome accession numbers, isolation source, genome completeness and contamination assessed by checkM and NCBI taxonomy for genomes included in the comparative study.

**Table S2:** Specific query sequences used for BLAST searches for lanthanide-dependent transporters in plasmids and lake sediment metatranscriptomes within IMG.

| **Name** | **Sequence** | **Reference** |
| --- | --- | --- |
| Lanmodulin (LanM) | MAFRLSSAVLLAALVAAPAYAAPTTTTKVDIAAFDPDKDGTIDLKEALAAGSAAFDKLDPDKDGTLDAKELKGRVSEADLKKLDPDNDGTLDKKEYLAAVEAQFKAANPDNDGTIDARELASPAGSALVNLIR | [1] |
| LanPepsy (LanP) | MVGKTLVVLSTFALAISSSLAIADHHFPKGKVSLETCLEAALKAKPGTVVKVEYKLEGETPVYEFDIESSDSTAWDVECDANTGKIVEIEQEVDSADHPLFKAKQKVSEAEARKTALAAHPGEIVEVEYEIEENGAASYEFDIKTKDGKEFKVEVDASTGKIVEANQEFYQIGKE | [2] |
| TonB-dependent receptor (LanA) | MSLTSIIKIESNQNATGSGQGQIAPFRMCLMTVAVLAAVNANAAKVDDRPPAVKLPPLEIIGEEPSQLEHIPGSGFVIDKTTLDRQGPLSTKDALRTIPGIHIVDEDVLGRRFNLGIRGLDPRRSVRTQLLEDGAPIQLAPYSDPSNHYIPTNKRIDRIEVLKGSGQIMYGPQTVGGAVNFVSAPIPEEFGGSISAAGGNNGYYDTHLRLGGTLDNVGLSLDYIRQESDGNRSGQHQEVDDLALKALIKIDDRQRLMLKGILTHEDADMGEAGLTSEMYRRNQRTNPLRNDSFEVRRYAGQALYEFDISDTMQFSTNIYGNHMFRESIRQANDSSQMNNCANRREPISADVAPTCGNEQRPRTYNVFGIEPKLVFMHDAFGLQSETTLGIRGHFEWADRERYVGNAGPRDTTQGRENNNHGRNRYQDNSLETQALSFFAQNRFFLGDFTVTPGVRVEHYYQDNINNIDGATESLVRTEALPGVGVTYNGIDNTTLFAGVHRGFAPGRIGDFVDPTKNILSQVEPELSLNYEAGVRTSPTPGVNLEMTYFRIDFENQIVEDITVEDTRFVNVGETVHQGVETGFRLDSNQLFGTDYNYYLTTSYTYLDAYFASNEARAGIVRDNRLPYAPEHLINANVGVETPWGLDIRFGIQSVSQQYVDIENTREENANGQEGIIPGYTVFNVSANYQVVKNVNVFMNGYNLSDKKFIASRVDGIHPGQGFQMMGGVKWTF | [3] |
| TonB-dependent receptor (LutH) | MLGSRSRISLIALLAAATASHAARAQEALPDIEIATAPEPGAKVTQSKLSTNPIILTRSDLDRDHAVSITDTLLRRNASISSSEVAGNPFQPDISYRGFSASPVPGTPQGLAVYQDGVRINEAFGDSVNFDLIPTVAIANGDIVSGNPMFGLNALGGALTLEMKNGFTWQGVEVDGRGGSYGRRFGSVQYGQKFDDLAVYLAFEGLGERGWRKRSGSSLLRGYGDIGYKGDKTEFHLNFTGAGTRLGNAAATPVEMLERQYNSVFTTPQSSNNDLLFVNLKGAYEADAHLKFNGNTYFRQFRQQHVDGNLADVEECGSDEQWLCQETDGFANEPSFQQSVLRDGNGKRIPSSVLNGAGVGSIDYTRVNSRSYGFALQGTYDAPVFGFANKLIVGASHDRQHSDFRGYSELGILNGNLAVGGTGLLYSNLVPEGGFQPVHVGGRNFYWGVYAHDTLEVTDKLTATAGLRFNSADIALFDHRGVSLNSAALYQRVNPVAGLTYTFLPELTVYGNYSEANRAPTALENGCSDRLHPCMIDNFLVSDPPLRQVVARTWETGLRGHHEFGGDYGRADWHAGLFRTDSADDILSLPSDVIQGRGYYANVGHTRRQGVEAGVTWRNDFLQVYADYALIDATFRSFLTLNSGDNPYADENGQIHVHPGNVLPSIPRHRVKVGFDYKVTPQWTFGLDYIFRTGVHLAGDEANLDRPLSSYGIVNLKTSYKVTDNIEIYSVVQNLFDRRYYSFGTYFETGSEAIGFLGLTDPRTLGPGAPLAAYGGVKVRF | [4] |

**Table S3:** The biochemically characterised Lanmodulin motifs and PepSY domains. The single regular expressions (REGEX) patterns were created based on the motif sequences.

| **Name of motif/domain** | **Sequence/Description** | **Reference** |
| --- | --- | --- |
| Mex-LanM | EF1 35 D P D K D G T I D L K E 46  EF2 59 D P D K D G T L D A K E 70  EF3 84 D P D N D G T L D K K E 95  EF4 108 N P D N D G T I D A R E 119 | [1] |
| Mex-LanM REGEX | [DN] P D [KN] D G T [IL] D [AKL] [KR] E | This study |
| Hans-LanM | EF1 34 N K D N D D S L E I A E 45  EF2 58 N P D G D T T L E S G E 69  EF3 83 N K D g D Q T L E M D E 94  EF4 107 D A N K D G K L T A A E 118 | [5] |
| Hans-LanM REGEX | [DN][KPA][DN][NGK]D[DTQG][STK]L[IET][ESMA][AGD]E | This study |
| Calmodulin EF-hand (PS00018) | DX[DNS]XXX[GP]XXXX[DE] | [6] |
| PepSY domain | The PepSY domain varies from 60–90 residues in length and is predicted to have an α/β fold. It often occurs as a single copy and in multiple domain architectures; this suggests that it is stable in isolation and is a true domain. | [7] |

**Table S4:** The composition of the concentrated ore

| **S/N** | **Rare earth element** | **Mass (%)** |
| --- | --- | --- |
| 1 | Lanthanum (La) | 16.6 |
| 2 | Cerium (Ce) | 37.3 |
| 3 | Praseodymiun (Pr) | 4.35 |
| 4 | Neodenyum (Nd) | 17.2 |
| 5 | Samarium (Sm) | 2.57 |
| 6 | Europium (Eu) | 0.13 |
| 7 | Gadolinium (Gd) | 2.12 |
| 8 | Terbium (Tb) | 0.31 |
| 9 | Dysprosium (Dy) | 1.74 |
| 10 | Holmium (Ho) | 0.34 |
| 11 | Erbium (Er) | 0.87 |
| 12 | Thulium TM | 0.12 |
| 13 | Ytterbium (Yb) | 0.58 |
| 14 | Lutetium (Lu) | 0.07 |
| 15 | Yttrium (Y) | 8.34 |
| 16 | Scandium (Sc) | 0.01 |

**Table S5:** Distribution of methanol dehydrogenase and lanthanide-dependent genes in methane-oxidising bacteria.

**Table S6:** Proteomics response of *Methylosinus trichosporium* OB3b to cerium and mixed lanthanide ore.

**Supporting Figures**

**
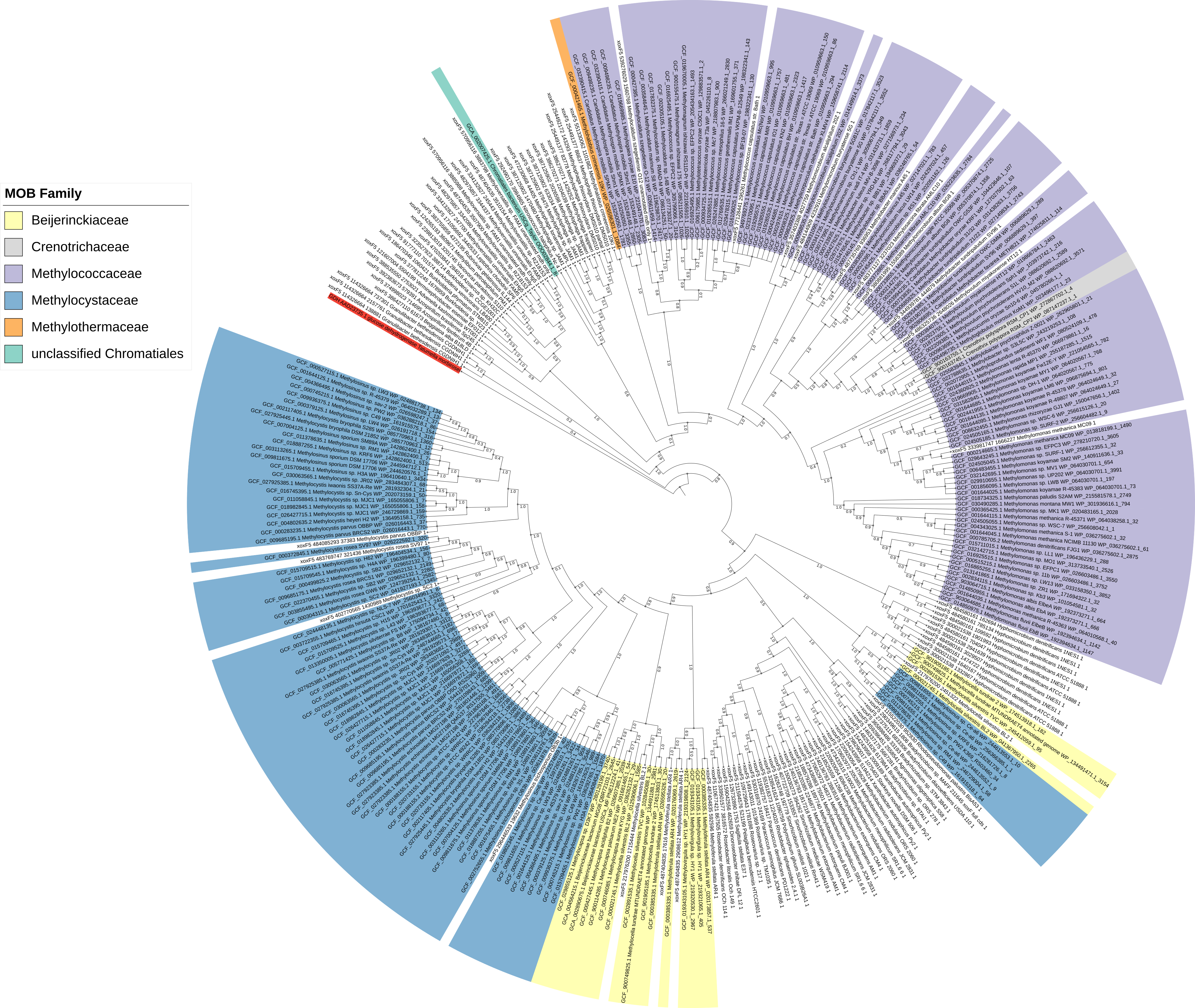
**

**Figure S1:** Circular phylogenetic tree showing XoxF5 sequences from the MOB genome database. The dark red-coloured leaf represents a glucose dehydrogenase used as an outgroup. Leaves without colored ranges are reference sequences. Bootstrap values (1,000 replicates) are shown at the nodes to one decimal place. The tree was annotated using iTOL.

**
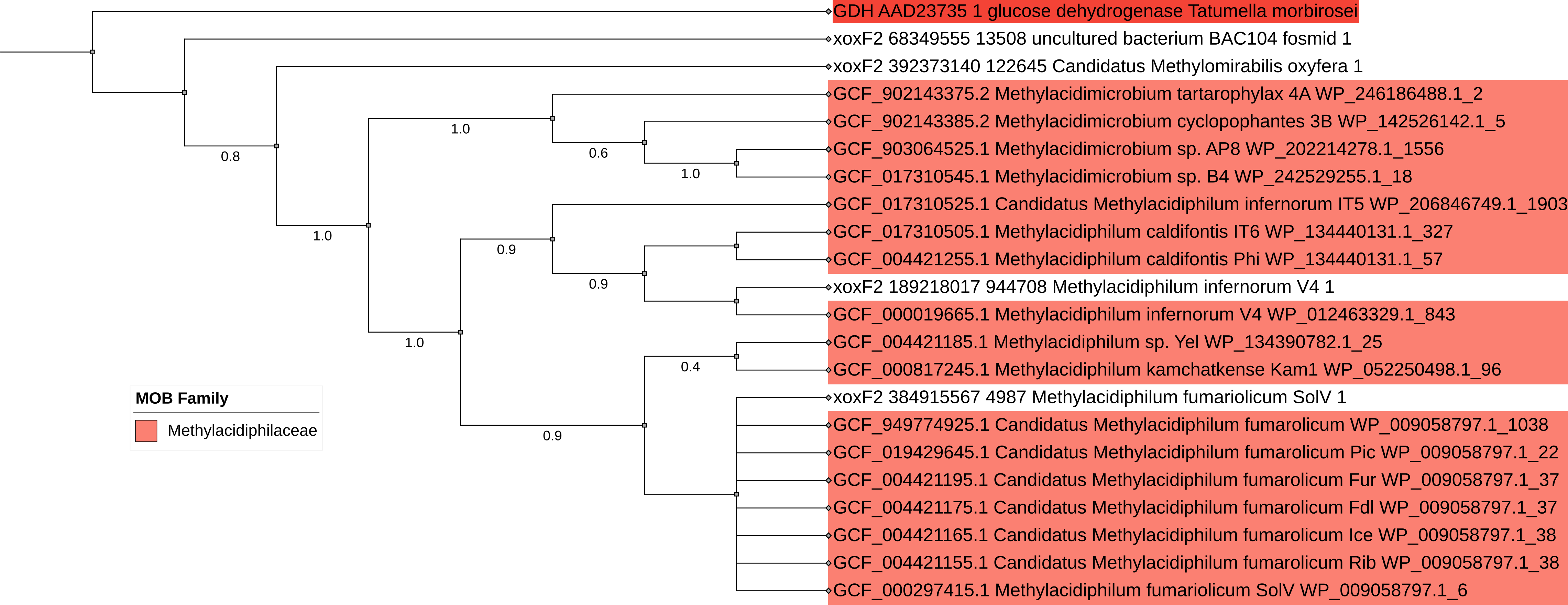
**

**Figure S2:** Rectangular phylogenetic tree showing XoxF2 sequences from the MOB genome database. The dark red-coloured leaf represents a glucose dehydrogenase used as an outgroup. Leaves without colored ranges are reference sequences. Bootstrap values (1,000 replicates) are shown at the nodes to one decimal place. The tree was annotated using iTOL.

**
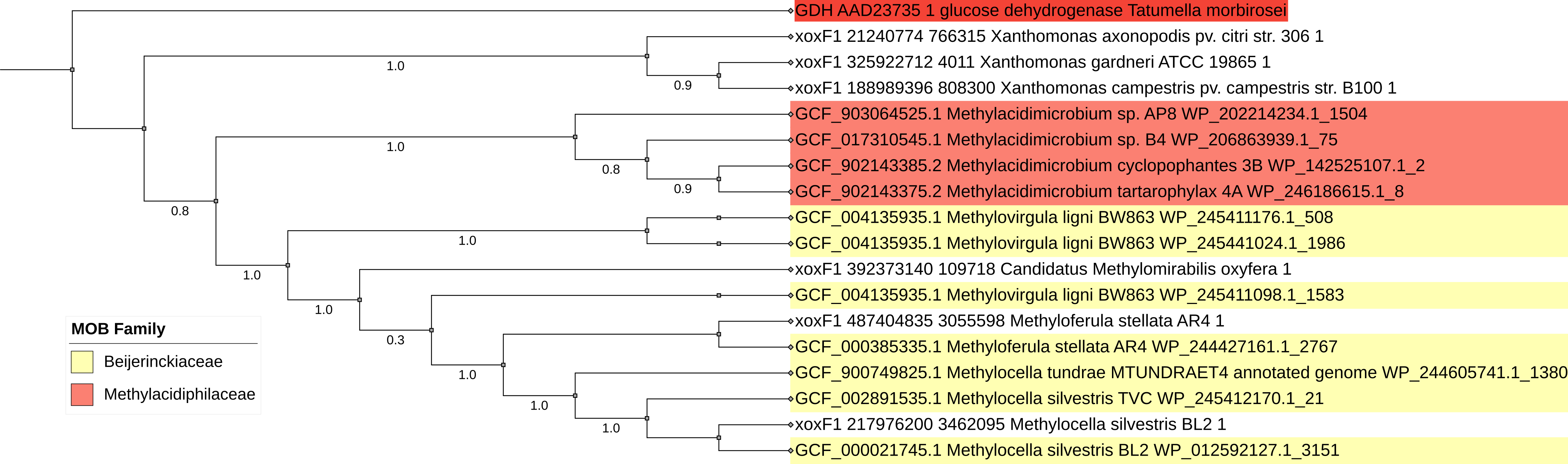
**

**Figure S3:** Rectangular phylogenetic tree showing XoxF1 sequences from the MOB genome database. The dark red-coloured leaf represents a glucose dehydrogenase used as an outgroup. Leaves without colored ranges are reference sequences. Bootstrap values (1,000 replicates) are shown at the nodes to one decimal place. The tree was annotated using iTOL.

**
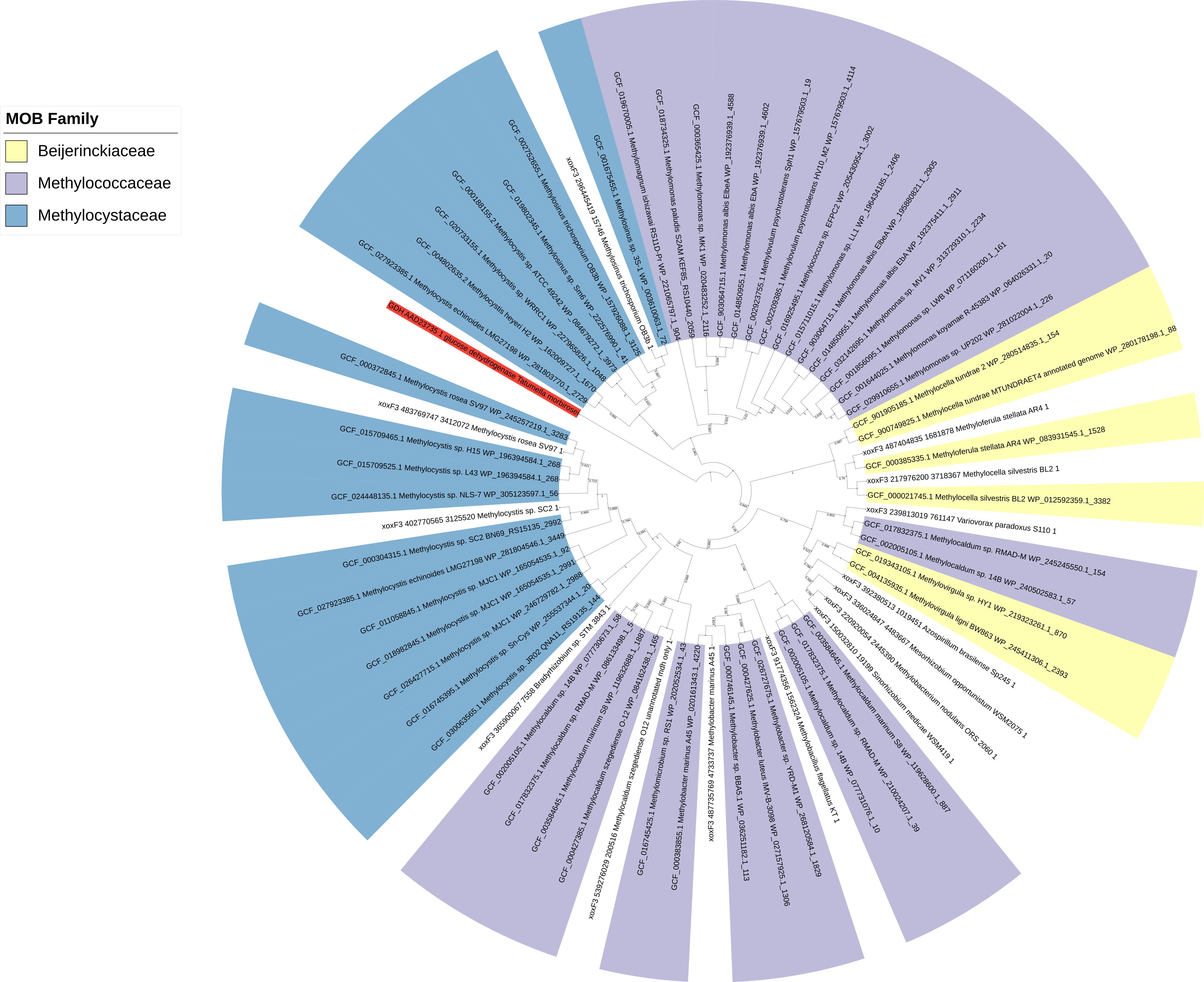
**

**Figure S4:** Circular phylogenetic tree showing XoxF3 sequences from the MOB genome database. The dark red-coloured leaf represents a glucose dehydrogenase used as an outlgroup. Leaves without coloured ranges are reference sequences. Bootstrap values (1,000 replicates) are shown at the nodes to one decimal place. The tree was annotated using iTOL.

**
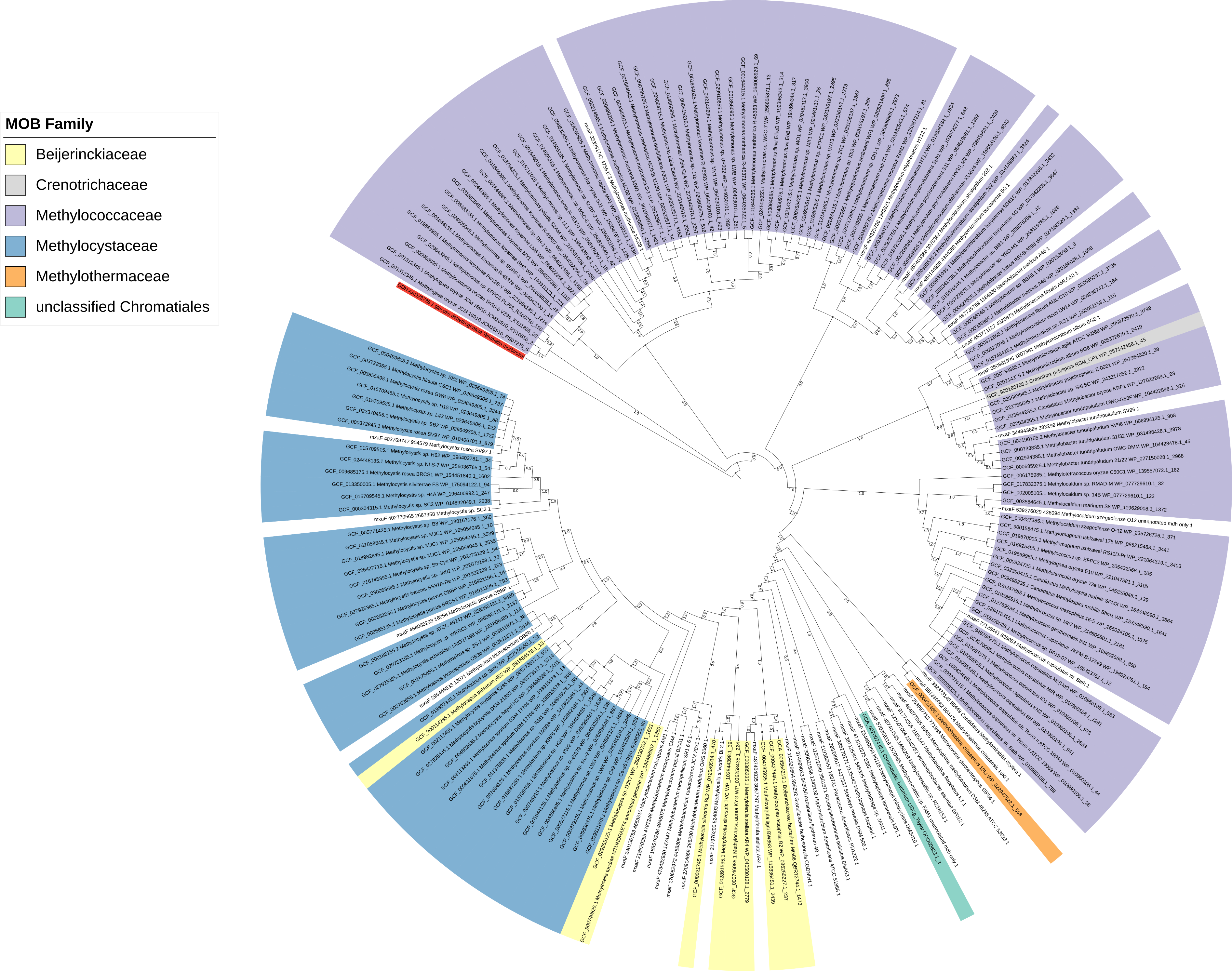
**

**Figure S5:** Circular phylogenetic tree showing MxaF sequences from the MOB genome database. The dark red-coloured leaf represents a glucose dehydrogenase used as an outgroup. Leaves without colored ranges are reference sequences. Bootstrap values (1,000 replicates) are shown at the nodes to one decimal place. The tree was annotated using iTOL.

**References**

1. Cotruvo JA, Featherston ER, Mattocks JA, Ho J V., Laremore TN. Lanmodulin: A Highly Selective Lanthanide-Binding Protein from a Lanthanide-Utilizing Bacterium. J Am Chem Soc. 2018;140:15056–61.

2. Hemmann JL, Keller P, Hemmerle L, Vonderach T, Ochsner AM, Bortfeld-Miller M, et al. Lanpepsy is a novel lanthanide-binding protein involved in the lanthanide response of the obligate methylotroph Methylobacillus flagellatus. Journal of Biological Chemistry. 2023;299:102940.

3. Yu Z, Groom J, Zheng Y, Chistoserdova L, Huang J. Synthetic methane-consuming communities from a natural lake sediment. mBio. 2019;10.

4. Shiina W, Ito H, Kamachi T. Identification of a TonB-Dependent Receptor Involved in Lanthanide Switch by the Characterization of Laboratory-Adapted Methylosinus trichosporium OB3b. Appl Environ Microbiol. 2023;89.

5. Mattocks JA, Jung JJ, Lin CY, Dong Z, Yennawar NH, Featherston ER, et al. Enhanced rare-earth separation with a metal-sensitive lanmodulin dimer. Nature 2023 618:7963. 2023;618:87–93.

6. Zhou Y, Yang W, Kirberger M, Lee HW, Ayalasomayajula G, Yang JJ. Prediction of EF-hand calcium-binding proteins and analysis of bacterial EF-hand proteins. Proteins: Structure, Function and Genetics [Internet]. 2006 [cited 2025 Jul 24];65:643–55. Available from: /doi/pdf/10.1002/prot.21139

7. Yeats C, Rawlings ND, Bateman A. The PepSY domain: A regulator of peptidase activity in the microbial environment? Trends Biochem Sci [Internet]. 2004 [cited 2025 Jul 16];29:169–72. Available from: https://pubmed.ncbi.nlm.nih.gov/15124630/
